# Modulating glutamate neurotransmission in *in vivo* models of Multiple Sclerosis: a narrative review

**DOI:** 10.3389/fimmu.2026.1811323

**Published:** 2026-08-10

**Authors:** Eleni Stamoula, Theofanis Vavilis, Ioanna Boskou, Olga Vampertzi, Nikoletta Floropoulou, Antonia Demopoulos, Rafaella Grigoriou, Mikhail Melnikov

**Affiliations:** 1 Department of Health Sciences, School of Life and Health Sciences, University of Nicosia, UNIC Athens, Athens, Greece; 2 Department of Dentistry, European University Cyprus, Nicosia, Cyprus; 3 Laboratory of Biology and Genetics, School of Medicine, Aristotle University of Thessaloniki, Thessaloniki, Greece; 4 School of Medicine, Aristotle University of Thessaloniki, Thessaloniki, Greece; 5 4^th^ Department of Pediatrics, School of Medicine, Faculty of Health Sciences, Aristotle University of Thessaloniki, Papageorgiou Hospital, Thessaloniki, Greece; 6 Laboratory of Neuroimmunology, Federal Center of Brain Research and Neurotechnologies of the Federal Medical-Biological Agency of Russia, Moscow, Russia

**Keywords:** EAE, excitotoxicity, glutamate neurotransmission, immunoexcitotoxicity, immunomodulation, *in vivo*, Multiple Sclerosis

## Abstract

Glutamate (Glu), the primary excitatory neurotransmitter of the central and peripheral nervous systems, plays essential roles in cognition, synaptic plasticity, and immune modulation. Its dysregulation is increasingly recognized as a component of neuroinflammation and neurodegeneration in multiple sclerosis (MS). In MS and its principal experimental model, experimental autoimmune encephalomyelitis (EAE), impaired Glu homeostasis leads to excitotoxicity, calcium overload, and oxidative damage, compounded by proinflammatory mediators such as TNF-α. This makes glutamatergic receptors, transporters, and related signaling pathways candidate therapeutic targets that warrant systematic preclinical evaluation. To assess the preclinical evidence base for agents targeting these pathways, we conducted a systematic literature search, we conducted a systematic literature search and comparatively assessed published *in vivo* studies examining the effects of glutamatergic agents in EAE models, applying predefined inclusion and exclusion criteria Across the reviewed EAE studies, glutamatergic agents were frequently reported to delay disease onset, alleviate clinical symptoms, attenuate neuroinflammation, and reduce demyelination, although effect sizes and reproducibility varied across models, dosing regimens and treatment timing. Collectively, these data indicate that pharmacological modulation of glutamatergic signaling can engage multiple facets of EAE pathophysiology. However, completed clinical trials of repurposed glutamatergic drugs (riluzole, amantadine, memantine, lamotrigine) have so far failed to demonstrate disease-modifying efficacy in MS, underscoring a marked translational gap.

## 1 Introduction

### 1.1 Glutamate transmission, receptors and metabolism

Glutamate (Glu) is the primary excitatory neurotransmitter in the central and peripheral nervous system (CNS and PNS), involved in learning, memory, cognition and synaptic plasticity under normal conditions (1, 2). The amount of glutamate is tightly regulated in the central nervous system. Glu plays an important signaling role in peripheral organs and tissues; the heart, kidney, intestine, lungs, muscles, liver, ovary, testis, bone and pancreas are all home to glutamate signaling, as well as the adrenal, pituitary and pineal glands (3). Various Glu receptors are expressed, not only from neuronal and glial cells, but also from peripheral non-neuronal tissues outside of the CNS; this further confirms the role of Glu as an extracellular signal mediator in the autocrine and/or paracrine system (4). Furthermore, Glu induces direct effects on most cells of the immune system functioning as an immunomodulator in the initiation and development of adaptive immune responses (3, 5).

In the brain, two major classes of Glu receptors have been described: ionotropic glutamate receptors (iGluR) and metabotropic glutamate receptors (mGluR). The former, subdivided into AMPA, NMDA and KA receptors, form ion channels that initiate Ca2+ and/or K+ influx and mediate fast excitatory glutamate responses (2, 3). The latter are G protein-coupled receptors and categorized into three groups: Group I, which contains mGlu1 and mGlu5 receptor subtypes, primarily activates phospholipase C. Group II and Group III consist of Glu2, mGlu3 receptors and mGlu4, mGlu6, mGlu7, mGlu8 receptors, respectively; these receptor groups work via a negative coupling mechanism (6). The mGluRs are found in pre- and postsynaptic neurons in synapses of the hippocampus, cerebellum and the cerebral cortex, as well as in other brain regions and in peripheral tissues. Glutamate can activate all its ionotropic and metabotropic receptors, yet there are many selective GluR agonists and antagonists. Activation of GluRs in the nervous system is responsible for the basal excitatory synaptic transmission and many forms of synaptic plasticity.

In the glutamatergic presynaptic terminal, vesicular glutamate transporters (VGLUTs) store Glu in synaptic vesicles; Glu is then released into the synaptic cleft via exocytosis with a Ca2+-dependent mechanism. However, a separate Ca2+-independent transport system is described in microglial cells that imports cystine (the oxidized dimer form of cysteine) into the cell in exchange for internal glutamate and constitutes the glutamate/cystine exchanger or X_c_^-^ antiporter system (2).

Upon delivery, under physiological conditions, glutamate transporters in the plasma membranes of neuronal cells rapidly clear the released glutamate and maintain low Glu concentrations in the synaptic cleft. This is achieved by Glu uptake via excitatory amino acid transporters (EAATs or XAG − system) expressed on astrocytes, oligodendrocytes and neurons; and via the high-affinity Na+ -dependent glutamate/aspartate/cystine transporter (XAG system) expressed mainly in astrocytes (5).

With the proper uptake of Glu by astrocytes surrounding the synaptic cleft, two biochemical pathways are then associated with its metabolism. In the first pathway, Glu can be converted into glutamine by the enzyme glutamine synthetase (GS), which remains as a precursor for Glu (7). In the second pathway, glutamate can be converted to the TCA cycle intermediate, α-ketoglutarate (α-KG), by three aminotransferase reactions, with aspartate aminotransferase (AAT) being the most active in brain cells. Apart from that, it can also undergo oxidative deamination into α-ketoglutarate via the enzyme glutamate dehydrogenase (GDH) (7). Thereby, it is evident that glutamate is closely associated with cellular energy metabolism.

### 1.2 Glu effects on T-cell mediated immunity

As already mentioned, glutamate affects most cells of the immune system. It has been reported that T-cells, neutrophils, dendritic cells (DCs), monocytes, macrophages, and microglia can produce glutamate (3). Furthermore, immune cells, especially T-cells, express a substantial amount of functional iGluRs and mGluRs, underlying the role of glutamate as a key immunomodulator in peripheral tissues (3, 5). The current paper will refer to the role of glutamate on T-cell mediated immunity.

Specifically, it has been reported that DCs release a significant quantity of glutamate during maturation and contact with T-cells. This is mediated by the DC-expressed Xc- antiporter (6). As detailed below, the released glutamate induces the proliferation of T-cells and the secretion of cytokines via specific GluRs, in addition to controlling immune responses. These are determined by the specific GluRs types expressed on the target T-cell, the T-cell type and subtype, the T-cell resting or activated state, and the presence or absence of other simultaneous stimuli besides glutamate (3).

It has been described that in normal resting T-cells, glutamate effectuates two vital functions via ionotropic AMPA GluR3; the first one is the adhesion to two major glycoproteins of the extracellular matrix (ECM), fibronectin and laminin, and the second one is chemotactic migration towards the key chemokine CXCL12/SDF-1 (3). After the T-cell is activated via its T-cell receptor (TCR), it produces and secretes a proteolytic enzyme, granzyme B that causes GluR3 degradation. As a result, activated T-cells do not express high levels of GluR3 on their surface (3). This elimination in pro-adhesive and pro-migratory effects of Glu on activated T-cells may happen when the T-cells have reached their target and need to remain in site, without any further adhesion or migration (3).

Furthermore, at physiological concentrations, Glu augments intracellular Ca2+ (iCa2+) in activated T-cells, via AMPA, NMDA and KA iGluRs. This is of great significance since the increase of iCa2+ contributes to various functions of activated T-cells, including proliferation (3).

Apart from iGluRs, mGluRs are also expressed on the surface of human T-cells. Specifically, mGluR5 (Group I) is expressed in naive T-cells and probably impairs T-cell proliferation when a non-specific antigen is presented (5). During T-cell activation, mGluR1 (Group I) expression is induced and leads to the attenuation of inhibitory mGluR5 effects, enhancement of Th1 (IL-2 and IFN-γ), and pro-inflammatory (IL-6 and TNF-α) cytokine secretion (5).

In conclusion, it is obvious that glutamate has a vast array of effects on immune cells’ functions, maintaining or limiting ongoing immune responses, but there are still mechanisms that remain unclear and require further investigation (3).

### 1.3 Glu transmission in MS/EAE

Multiple Sclerosis (MS) is an autoimmune disease of the CNS that involves immune attack against CNS antigens mediated through activated CD4+ myelin reactive T-cells, with a possible contribution by B-cells. The comprehension of MS has been significantly subserved by the study of experimental autoimmune encephalomyelitis (EAE). EAE is an animal model of CNS inflammatory demyelination that can be induced by peripheral immunization with myelin protein components, commonly in rodents (8). EAE is widely considered the most representative *in vivo* model simulating Relapsing-Remitting Multiple Sclerosis (RRMS) (9, 10). Concisely, for the development of EAE, mice are immunized against myelin peptides such as myelin oligodendrocyte glycoprotein (MOG), proteolipid protein (PLP) and myelin basic protein (MBP) (11). It is acknowledged that MS and EAE share common cytological and molecular features that include the infiltration of macrophages and CD4+ T-cells and the activation of a Th1-like immune response (12). These cytological events, along with produced proinflammatory cytokines, such as IFN-γ, TNF-α and IL-6 and antibodies reactive to myelin, result in multifocal demyelination and axonal dystrophy (13). The main clinical features observed in MS and EAE are paralysis, ataxia, visual impairment, and relapses and remissions. Another similarity between EAE and MS is a genetic predisposition, such as MHC-linked susceptibility and increased female susceptibility (14).

Additionally, the pathology of MS includes both demyelination and axonal loss; the latter appears to be strongly associated with the disease’s disability progression (15). MS is categorized into two phases: an inflammatory relapsing remitting (RR) phase and a degenerative secondary-progressive (SP). The RR phase is mainly characterized by microglial activation and cellular immunity, while the SP phase involves humoral immunity, oxidative stress and excitotoxicity (15) Therefore, it is biologically plausible that glutamate homeostasis is involved in MS in multiple ways, including T-cell autoimmune responses and oxidative glutamate toxicity (6).

As previously mentioned, Glu induces pro-adhesive and pro-migratory effects on naive T-cells via iGluR AMPA GluR3, under normal concentrations (3). It has been proven that the expression of GluR3 in T-cells of MS patients is upregulated during relapse of the disease. As a result, Glu can increase proliferation and chemotactic migration of the MS autoreactive T-cells. Based on this, glutamate receptor antagonists could eliminate the unwanted effects of glutamate on autoimmune T-cells (3).

Glutamate excitotoxicity is closely related to autoimmune neuroinflammation (2) To begin with, excitotoxicity is defined as the pathological process by which nerve cells are damaged or killed by excessive glutamate stimulation (2). This inordinate effect of Glu is a result of both increased release of Glu and upregulation of its receptors that have been reported in MS patients. In the context of neuroinflammation, activated glia and peripheral immune cells that cross the blood-brain barrier (BBB) produce and secrete cytokines. These pro- inflammatory cytokines are involved in the dysfunction of excitatory amino acid transporters (EAAT), which has been reported in MS patients and leads to higher extracellular glutamate concentrations. The glutamate/cystine exchanger (system x_c_^-^ antiporter) is also overexpressed by microglia and astrocytes, increasing the amount of extracellular Glu further. Thus, glutamate binds to ionotropic receptors mainly via NMDA, which is permeable to calcium, and induces a massive influx of extracellular Ca2+. Through different pathways, including mitochondrial dysfunction, production of reactive oxygen species (ROS) and reactive nitrogen species, intracellular Ca2+ overload results in degeneration and neuronal cellular death (2).

Regarding proinflammatory cytokines, TNF-α is mentioned as the primary neurotoxic molecule in progressive forms of MS. TNF-α can activate caspase-dependent apoptosis and enhance the production of ROS via autocrine and paracrine signaling inducing cell death and neurodegeneration (2). Besides glutamate and TNF-α, other neurotoxic substances are also produced by the reactive neuroglia, including IL-1β, prostaglandins, ROS, nitrogen species, S100B (2).

In conclusion, Glu receptors and transporters, together with other mediators of neuroinflammation such as cytokines, represent mechanistically plausible targets for future MS therapeutics; whether this mechanistic rationale can be translated into clinical benefit remains to be demonstrated, as furtherly analyzed in discussion section. It should also be acknowledged that the EAE literature on glutamatergic modulation is methodologically very heterogeneous, and this heterogeneity directly dictates how the results summarized in tables provided can be interpreted. Studies differ in the encephalitogenic antigen used (MOG35–55, MBP, PLP139–151 or whole spinal cord homogenate), the species and strain employed (Lewis rats, C57BL/6, SJL, NOD and ABH Biozzi mice), and the adjuvant/pertussis toxin regimen, each of which produces a distinct immunopathological phenotype ranging from acute monophasic to chronic-progressive and relapsing–remitting disease (10, 11, 16). Within a single agent class, doses can differ by one to two orders of magnitude (for example, memantine 1.5–60 mg/kg, MK-801 0.15–0.6 mg/kg), and treatment is variously initiated prophylactically, at immunization, at first clinical signs or only during the chronic phase. Outcome assessment is similarly inconsistent across studies, combining different clinical scoring scales, histological endpoints and behavioral tests. Compounding this, systematic appraisals of the preclinical MS literature have shown that randomization, blinded outcome assessment and *a priori* sample-size calculations are reported in only a minority of EAE studies, and that experiments incorporating these safeguards yield significantly smaller effect sizes than those that do not (17, 18). Accordingly, the apparent consistency of beneficial effects across the agents reviewed here should be interpreted with great caution, and individual comparisons between compounds, doses and disease phases must be made with these methodological caveats explicitly in mind.

## 2 Methods

We conducted a literature search through Pubmed, Scopus and Google Scholar for *in vivo* studies on the prognosis or progression of the EAE model of MS combined with glutamatergic drugs or agents administration. Our search included glutamatergic agents that act on different sites on the glutamatergic neurotransmission. Namely, agents that act on Glu transporters, on Glutamate Receptors (ionotropic, metabotropic) and agents that interfere with glutamate synthesis, metabolism, uptake and release and chronologically covered a time period from inception dating back to 199 5until the 1st of March 2025.

Certain keywords were used:

1. [(NMDA agonist) OR (NMDA antagonist) AND (EAE)].
2. [(AMPA agonist) OR (AMPA antagonist) AND EAE].
3. [(Glutamate transporters) AND EAE].
4. [Glutamate Synthesis OR Metabolism OR Release AND EAE].

All articles retrieved through the databases were imported to Zotero after being independently screened for eligibility and duplicity. We applied certain inclusion/exclusion criteria in our literature search. Only *in vivo* original research papers were included, published in English, describing the use of Glutamatergic agents as a monotherapy or combination treatments exclusively on the EAE model. Articles where only the abstract was available, were not included.

## 3 Results

### 3.1 NMDA and metabotropic glutamate receptor agents in EAE

#### 3.1.1 NMDA receptor antagonists

##### 3.1.1.1 Memantine

Memantine is a non-competitive NMDA receptor antagonist that has been evaluated for its neuroprotective roles in EAE models. Experiments with intraperitoneal administration of the compound (10–20 mg/kg) in guinea pig spinal cord–induced EAE in Lewis rats were conducted. In those experiments, a reduction in neurological deficits and an improvement in clinical scores were observed. No statistically significant differences were detected in CNS inflammation levels (19). Along the same line of research, Paul et al. showed that both semi prophylactic (60 mg/kg) and therapeutic (40–60 mg/kg) treatment protocols attenuated neurological symptoms, reduced BBB permeability, decreased lesion development, decreased inflammatory infiltrate intensity, and improved neurovascular function in the cerebellum (20).

Experiments in similar models of EAE in Lewis rats confirmed that memantine (60 mg/kg) reduced weight loss, neurological deficits, and disease severity. Increased MBP and myelin-associated glycoprotein (MAG) mRNA expression was also detected (21). It is to be noted, though, that memantine failed to exhibit a protective effect on myelin ultrastructure. Further work from the same group confirmed previous clinical results and established that memantine can provide neuroprotection through a decrease in oxidative stress levels. This was accompanied by upregulation of superoxide dismutase 1 (SOD1) expression and preservation of the enzyme’s activity (22). In another series of experiments, Sulkowski et al. demonstrated that memantine (60 mg/kg) reduced paralysis and disease progression in EAE, decreased brain glutamate levels, and downregulated ionotropic glutamate receptor subunits mGluR1 and mGluR5 (23–25). In one of those studies, an anti-inflammatory role of memantine in EAE was proposed, as it was found to markedly decrease proinflammatory cytokines and chemokines (IL-1β, IL-6, fractalkine, MIP-1, MIP-3, and TNF-α) (24). These effects also appear to be reflected in a separate study, which demonstrated that memantine slowed disease progression and attenuated demyelination (26). In other EAE models, however, memantine at 1.5 mg/kg failed to show any effects on disease progression (27).

Amantadine, a structurally similar NMDA receptor antagonist, was examined alongside memantine in several studies. Amantadine (100 mg/kg) was reported by Dąbrowska-Bouta et al. to produce effects comparable to memantine, both at the clinical and biochemical levels (21). A series of experiments by Sulkowski et al. utilizing the same dose further validated these observations, showing comparable actions to memantine (23–25). Amantadine also appears to downregulate NMDA receptor mRNA (25).

##### 3.1.1.2 MK-801 (Dizocilpine)

The effect of another non-competitive NMDA receptor antagonist, MK-801, was investigated in EAE Lewis rat and ABH Biozzi mouse models. MK-801 (0.15–0.6 mg/kg) produced dose-dependent reductions in neurological deficits and clinical disease severity in acute EAE (28). Specifically, at 0.15 mg/kg it appeared to attenuate EAE-associated cerebrovascular disruption and neurological deficits, whereas at 0.3 mg/kg it also delayed disease onset (28). More recent experiments, in which MK-801 was administered at 0.3 mg/kg, demonstrated decreased levels of prostaglandins PGE2 and PGD2 in the cerebral cortex and spinal cord, respectively (29). Apart from delaying the acute phase and reducing clinical scores and neurological deficits, MK-801 has also been found to normalize electrophysiological findings, including a reduction in spontaneous excitatory postsynaptic current (sEPSC) frequency and an increase in paired-pulse ratio (PPR) when administered at 6.5–30 μM (30). Another study comparing MK-801 (150 μg/kg) with other NMDA antagonists in both chronic progressive (NOD mice) and relapsing–remitting (SJL mice) EAE models failed to find any effects on disease progression (27).

##### 3.1.1.3 Selective NMDA receptor subunit antagonists

Wang et al. investigated (R)-ketamine, a GluN2B/NMDA receptor antagonist, demonstrating that treatment (10 mg/kg) reduced clinical scores and weight loss, as well as BBB damage and demyelination, in MOG-induced EAE. Furthermore, (R)-ketamine exerted immunomodulatory effects by reducing inflammatory cell infiltration and microglial activation (31).

Highly selective GluN2B/NMDA receptor antagonists, such as RO25-6981, have been examined in MOG-induced EAE in C57BL/6 mice. Treatment with this agent resulted in attenuation of weight loss and reduced clinical scores at a dosage of 25 mg/kg, whereas reductions in inflammation, axonal degeneration, and demyelination were noted at 10 and 25 mg/kg (26). At the 25 mg/kg dose, this compound displayed superior neuroprotective actions compared with memantine.

##### 3.1.1.4 Other NMDA receptor antagonists with mixed pharmacology

Fullerene ABS-75, a novel NMDA antagonist and antioxidant, was evaluated by Basso et al. and demonstrated efficacy against chronic EAE, with delayed disease progression, reduced clinical scores, and immunomodulatory actions, without concurrent deleterious effects on memory function, whereas it appeared to have no effect in relapsing EAE models (27).

Dextromethorphan (DXM), an NMDA channel blocker that, among other actions, can also exert σ1 agonism and serotonin–norepinephrine reuptake inhibition, has been studied at doses of 0.1–10 mg/kg in MOG-induced EAE. Treatment at 0.1 mg/kg did not demonstrate statistically significant results in severe EAE, but in moderate cases it appeared to decrease clinical scores, inhibit demyelination and axonal loss, and beneficially modulate immunological responses, oxidative stress, and inflammation (32).

Another compound with mixed pharmacology, 2-benzofuranyl-2-imidazoline (2-BFI), an NMDA inhibitor and imidazoline 2 (I2) receptor agonist, demonstrated beneficial effects at 20 mg/kg in MOG-induced EAE. These effects included decreased BBB permeability, reduced infiltration of inflammatory cells, and decreased neurological deficits, along with a concurrent reduction in loss of occluding proteins and activation of the NR1–ERK pathway (33).

#### 3.1.2 Metabotropic glutamate receptor agents

##### 3.1.2.1 Group I mGluR modulators (mGlu1 and mGlu5)

The selective mGlu1 antagonist LY367385 and the selective mGlu5 antagonist MPEP have been studied in great extent. Dąbrowska-Bouta et al. reported that although both compounds (10 mg/kg each) did not produce significant clinical improvements in EAE, they increased the mRNA expression levels of MBP, MAG, and MOG, while decreasing CNPase mRNA expression (21). These findings align with three experiments by Sulkowski et al., who confirmed that LY367385 and MPEP failed to yield notable clinical or immunomodulatory effects comparable to those observed with amantadine and memantine. However, they did observe an upregulation of mGluR1 and mGluR5 mRNA and protein levels following treatment (23–25). In contrast, Fazio et al. showed that the mGlu1 positive allosteric modulator RO0711401 (10 mg/kg) enhanced motor coordination and alleviated symptoms in MOG-induced EAE in C57BL/6 mice. Meanwhile, mGlu5 and mGlu1R antagonists either showed no significant benefit (MPEP, SIB-1757) or worsened motor performance (JNJ16259685) (34).

##### 3.1.2.2 Group II mGluR modulators (mGlu2/3)

LY379268, an mGlu2/3 receptor agonist, was evaluated against MOG-induced EAE at a dose range of 0.01–1 mg/kg, showing reductions in clinical scores. Mechanistically, LY379268 inhibited glutamate release from cortical and spinal cord synaptosomes, indicating presynaptic modulation of glutamate exocytosis; these effects were prevented by coadministration of the mGlu2/3 receptor agonist LY341495 (35).

##### 3.1.2.3 Group III mGluR modulators (mGlu4)

L-2-amino-4-phosphonobutyric acid (L-AP-4), an mGlu Group III agonist, reduced clinical scores and improved recovery rates in MBP-induced EAE in Lewis rats. Treatment decreased neuroinflammatory cytokine RANTES mRNA expression in the cerebellum and spinal cord and reduced MHC-II+ and CD4+ cells at 27 days post-immunization (36). Clinically, it increased the rate of recovery and decreased clinical scores.

Comparable effects appear to be exerted by mGlu4 positive allosteric modulators such as ADX88178 and PHCCC at 30–60 mg/kg and 3 mg/kg, respectively. ADX88178 produced reductions in clinical scores with sustained long-term therapeutic effects in MOG-induced EAE and also increased anti-inflammatory cytokines (IL-10, TGF-β) and indoleamine 2,3-dioxygenase 1 (IDO1) expression, suggesting immunomodulatory mechanisms (37). In this experiment, PHCCC appeared to reduce cAMP levels in DCs.

Gammon et al. further investigated PHCCC, another mGlu4 positive allosteric modulator, comparing a soluble formulation with a polymeric nanoparticle (NP) delivery formulation. NP-encapsulated PHCCC (3 mg/kg) significantly reduced clinical scores and disease severity and delayed disease onset. From an immunological point of view, the NP formulation decreased T-cell proliferation, inflammatory cytokines, and Th17 cells, while increasing regulatory T-cells and suppressing DCs activation (38).

The above results are presented in **Table 1**.

**Table 1:** Comparative assessment of *in vivo* studies on the effects of NMDA metabotropic acting agents on disease scores and progression.

Study	Drug and dosage	Induction of EAE protocol	Signs of EAE	Preventive or symptomatic treatment (drug administration)	Study design	(Species) age/gender/weight	Methods	Clinical results	Biological results
Wallstrom et al., 1996 (19)	Memantine (NMDAR antagonist) 10 and 20 mg/kg	Immunization in both hind footpads with a total of 50 mg guinea pig spinal cord, 2 mg Mycobacterium tuberculosis, 100 μl Freund’s incomplete adjuvant	Onset of clinical signs 9–11 dpi	(i.p.) administration on 7,9,11 and 13 dpi	3 groups • Control(saline) • 10 mg/kg • 20 mg/kg	Male Lewis rats Weight 310–370 and 200-260)	Immunohistochemistry Single cell assay In situ hybridization	↓ neurological deficits ↓ clinical score	No statistically significant difference in CNS inflammation
Paul et al., 2002 (20)	Memantine 20, 40,60 and 80 mg/kg	Inoculation with encephalitogenic emulsion, consisting of equal parts of guinea pig spinal cord, sterile phosphate-buffered saline (PBS), and incomplete Freund’s adjuvant was prepared and supplemented with 10 mg/ml Mycobacterium tuberculosis H37Ra. Each rat received 0.1 ml of inoculum per hind footpad.	Onset of clinical signs 10 dpi	(p.o.) administration a)semiprophylactic treatment 40,60,80 mg/kg for 6 days, beginning on 7 dpi b)therapeutic treatment 20,40,60,80 mg/kg on 10 and 11 dpi	a) semiprophylactic treatment 5 groups • Normal (n=12) • Vehicle (n=18) • 40 mg/kg(n=6) • 60 mg/kg(n=6) • 80 mg/kg(n=6) b) therapeutic treatment 6 groups • Normal (n=10) • Vehicle (n=20) • 20 mg/kg(n=5) • 40 mg/kg(n=6) • 60 mg/kg(n=7) • 80 mg/kg(n=7)	Male Lewis rats, 7 to 9 weeks old	Histological assessment Quantitation of BBB Integrity Labeling of Red Blood Cells with 111In-tropolonate Determination of BBB Permeability Corticosterone Radioimmunoassay	a) semiprophylactic treatment 60 mg/kg ↓ neurological symptoms b)therapeutic treatment 40 and 60 mg/kg ↓ neurological deficits	a) semiprophylactic treatment 60 mg/kg ↓ BBB permeability ↓ intensity of inflammatory infiltrates ↓ lesion development b) therapeutic treatment 40 and 60 mg/kg ↑ neurovascular function in cerebellum ↓ BBB permeability
Dabrowska-Bouta et al., 2015 (21)	Amantadine (NMDAR antagonist) 100 mg/kg Memantine 60 mg/kg LY367385 (selective mGlu1 antagonist) 10 mg/kg MPEP (selective allosteric mGlu5 antagonist) 10 mg/kg	Immunization (s.c.) in both hind feet with 100 µl of inoculum containing guinea pig spinal cord homogenate emulsified in Freund’s complete adjuvant (CFA) containing 5.5 mg/ml Mycobacterium tuberculosis H37Ra	Onset of clinical signs before 12 dpi Acute phase of EAE 12 dpi	Administration (i.p)once daily for 7 consecutive days, from 5 to 11 dpi	6 groups (n=12/group) • Control • EAE • EAE+amantadine • EAE+Memantine • EAE+LY367385 • EAE+ MPEP	Female Lewis rats Weight 200g	Real time PCR Electron microscopy	Amantadine/Memantine ↓ weight loss ↓ neurological deficits ↓ severity of EAE ↓ duration of disease LY367385/MPEP Statistically insignificant results	↑ MBP mRNA (all drugs) ↑ MAG mRNA(all drugs) ↑ MOG mRNA (LY367385&MPEP) ↓ CNPase mRNA (all drugs) None of the drugs had protective effect on myelin ultrastructure
Dabrowska -Bouta et al., 2021 (22)	Memantine 60 mg/kg	Immunization (s.c.) in both hind feet with an inoculum containing guinea pig spinal cord homogenate emulsified in Freund’s complete adjuvant containing 5.5 mg/mL Mycobacterium tuberculosis H37Ra	Onset of clinical signs 10 dpi	Administration (i.p) once daily from 5–11 dpi (therapy started at an early asymptomatic stage of EAE)	8 groups (n=8/group) • control (healthy) • EAE 4 dpi • EAE 12 dpi • EAE 20 dpi. • EAE 25 dpi. • EAE 12 dpi. + memantine • EAE 20 dpi. + memantine • EAE 25 dpi. + memantine	Female Lewis rats weight 190–200 g	Measurement of Lipid Peroxidation Measurement of the Level of Sulfhydryl Groups Western Blot Analysis Measurement of SOD’s Activity	↓ clinical score ↓ duration of disease	↓ oxidative stress ↑ levels of –SH groups protein/non protein ↑ SOD1 enzyme expression Prevented loss of SOD activity
Sulkowski et al., 2014 (23)	Amantadine 100 mg/kg Memantine 60 mg/kg LY367385 10 mg/kg MPEP 10 mg/kg	Immunization (s.c.) in both hind feet with 100 µl of inoculum containing guinea pig spinal cord homogenate emulsified in Freund’s complete adjuvant (CFA) containing 5.5 mg/ml Mycobacterium tuberculosis H37Ra	Onset of clinical signs 10 dpi Experiments performed in Acute phase (12 dpi)	(i.p.) drug administration between 5 and 11 dpi. (asymptomatic phase of disease)	6 groups • control • EAE • EAE+amantadine • EAE+Memantine • EAE+LY367385 • EAE+ MPEP	female Lewis rats 200 g	[^3^H] glutamate transport assay Real time PCR ^3^H MK-801 binding assay Electron microscopy	Amantadine/Memantine ↓ weight loss ↓ neurological deficits ↓ severity of EAE ↓ duration of disease LY367385/MPEP Statistically insignificant results	Amantadine/Memantine ↓ the EAE elevated levels of Glu transporter EAAC-1 Inhibited MK801 binding ↓ Glu uptake and release LY367385/MPEP Statistically insignificant results
Sulkowski et al., 2013 (24)	Amantadine 100 mg/kg Memantine 60 mg/kg LY367385 10 mg/kg MPEP 10 mg/kg	Immunization (s.c.) in both hind feet with 100 µl of inoculum containing guinea pig spinal cord homogenate emulsified in Freund’s complete adjuvant (CFA) containing 5.5 mg/ml Mycobacterium tuberculosis H37Ra	Onset of clinical signs 10 dpi Experiments performed in Acute phase (12 dpi)	Administration (i.p) once daily from 5–11 dpi (asymptomatic phase of disease)	6 groups • Control • EAE • EAE+amantadine • EAE+Memantine • EAE+LY367385 • EAE+ MPEP	female Lewis rats 190-200g	Rat cytokine array panel A Confocal microscopy Western blot analysis Real time PCR Immunohistochemistry Fluorescence microscopy	Amantadine/Memantine ↓ weight loss ↓ neurological deficits LY367385/MPEP Statistically insignificant results	Amantadine/Memantine ↓ ↓ cytokines and chemokines (IL-1β, IL-6, Fractalkine, MIP-1,MIP-3,TNF-α) LY367385/MPEP Statistically insignificant results In all treated groups activation of microglial cells
Sulkovski et al., 2013 (25)	Amantadine 100 mg/kg Memantine 60 mg/kg LY367385 10 mg/kg MPEP 10 mg/kg	Immunization (s.c.) in both hind feet with 100 µl of inoculum containing guinea pig spinal cord homogenate emulsified in Freund’s complete adjuvant (CFA) containing 5.5 mg/ml Mycobacterium tuberculosis H37Ra	Onset of clinical signs 10–11 dpi	Administration (i.p) once daily from 7–11 dpi Experimental observations in different time points 12,20,25 dpi and early(4–8 dpi)/late (12–25 dpi)phase	7 groups control • EAE • EAE+amantadine • EAE+Memantine • EAE+LY367385 • EAE+ MPEP • EAE+combination drug treatment	female Lewis rats Weight 180–200 g	Western blot analysis Real time PCR	Amantadine/Memantine ↓ neurological deficits ↓ severity of EAE ↓ duration of disease LY367385/MPEP Statistically insignificant results Combination treatment (NMDA/mGluR antagonists) Statistically insignificant results	Amantadine/Memantine ↓ Glu release ↓ the EAE elevated mGluR1 mRNA ↓ the EAE elevated mGluR5 mRNA ↓ the EAE elevated mGluR5 protein Amantadine ↓ NMDA mRNA LY367385 ↑ mGluR1 mRNA/protein MPEP ↑ mGluR5 mRNA/protein
Farjam et al., 2014 (26)	Memantine 20 mg/kg RO25-6981 (selective GluN2B/NMDAR antagonist) 3, 10 and 25 mg/kg	Inoculated with 250 μg MOG emulsified in CFA. MOG was dissolved in 100 μL phosphate buffered saline (PBS) mixed with 100 μL of CFA supplied with 4 mg/mL heat-killed Mycobacterium tuberculosis. Mixture was subcutaneously injected in the flank of mice under light ether narcosis. Subsequently, 500 ng pertussis toxin was dissolved in 200 μL PBS and administered intraperitoneally (i.p.) immediately and 48 hours later	Onset of clinical signs 11–12 dpi	(i.p) once daily 12–15 dpi	6 Groups • Sham (No EAE)-Control(PBS)(n=10) • EAE+PBS (n=10) • EAE+Memantine (20 mg/kg) (n=10) • EAE+ RO25-6981 (3 mg/kg) (n=9) • EAE+ RO25-6981 (10 mg/kg) (n=10) • EAE+ RO25-6981 (25 mg/kg) (n=9)	Female C57BL/6 mice 9–12 weeks old weight 18-22g	Clinical score assessment Immhnohistochemistry	RO25-6981 (25 mg/kg) ↓ weight loss ↓ neurological score RO25-6981 (10&25 mg/kg) ↓ ↓ inflammation ↓ axonal degeneration ↓ demyelination RO25-6981 (25 mg/kg) > Memantine (superiority in neuroprotective efficacy) Memantine ↓ disease progression ↓ demyelination	
Bolton et al., 2013 (29)	Dizocilpine maleate ((+) MK-801) (noncompetitive NMDA antagonist) 0.3 mg/kg	Immunization in both flanks, 0.15 mL of inoculum containing pooled, lyophilized syngeneic spinal cord, solubilized in sterile phosphate-buffered saline (PBS), and incomplete Freund’s adjuvant, supplemented with Mycobacterium tuberculosis H37Ra and Mycobacterium butyricum.	Onset of clinical signs 17 dpi	(i.p.) once daily for 3 days (beginning from initial weight loss)	4 groups • Normal • undosed • vehicle • (+)MK-801	Male ABH Biozzi mice 7–9 weeks	Isolation and quantitation of PGs		↓ PGE2 (cerebral cortex) ↓ PGD2 (spinal cord)
Bolton et al., 1997 (28)	MK-801 0.15, 0.3, 0.6 mg/kg	Immunization with emulsion comprising equal parts of guinea pig spinal cord, sterile PBS and incomplete Freund’s adjuvant, supplemented with 10 mg ml21 Mycobacterium tuberculosis H37Ra	Onset of clinical signs 10 dpi	*Preventive treatment* (i.p.)once daily, doses 0,15 & 0,3 mg/kg for 6 days, beginning 7 dpi *Therapeutic treatment* (i.p.)once daily, doses 0,15 & 0,6 mg/kg for 3 days, beginning 10 dpi(initial weight loss)	Preventive treatment • Vehicle (n=11) • MK-801 0,15 mg/kg (n=6) • MK-801 0,3 mg/kg(n=6) Therapeutic treatment • Vehicle (n=5) • MK-801 0,3 mg/kg (n=6) • MK-801 0,6 mg/kg(n=6)	Male Lewis rats, weight 200–250 g	Corticosterone Radioimmunoassay Light microscopy Labeling of red blood cells with 111In-tropolonate Evaluation of BBB integrity assay	MK-801 0,15 mg/kg ↓ neurological deficits ↓ cerebrovascular disruption MK-801 0,3 mg/kg ↓ ↓ BBB dysfunction ↓ weight loss ↓ neuroendothelial disruption ↓ lesion formation ↓ clinical score Delayed disease onset	
Basso et al., 2008 (27)	Fullerene ABS-75 (NMDA antagonist -Antioxidant) 30 μg/kg Memantine 1.5 mg/kg MK-801 150 μg/kg	A) chronic progressive EAE (NOD EAE) Immunization (s.c.) with 150 μgof MOG35–55 peptide in 4 mg/ml CFA. Pertussis toxin was given i.v. (150 ng per mouse) at the time of immunization and 48 h later B) SJL EAE (relapsing-remitting SJL model) Immunization(s.c.) with 50 μg of PLP131–151 peptide in 4 mg/ml CFA. Pertussis toxin was given i.v. (150 ng per mouse) at the time of immunization and 48 h later C) monophasic relapsing C57BL/6J (Acute EAE C57BL/6J) Immunization (sc) with 150 μg of MOG35–55 peptide in 4 mg/ml CFA. Pertussis toxin was given i.v.	Onset of clinical signs 10–11 dpi (in all groups)	A)chronic progressive EAE (NOD EAE) I experiment 30 μg/kg ABS-75 (i.p.)daily beginning on 20 dpi II experiment 30 μg/kg ABS-75 & Memantine 1,5 mg/kg (i.p.) daily beginning on 19 dpi B) SJL EAE 30 μg/kg ABS-75 & Memantine 1,5 mg/kg (i.p.) daily beginning on 17 dpi C) Acute EAE C57BL/6J (ip) ABS-75 30 μg/kg beginning day 1 before MOG immunization and daily	A)chronic progressive EAE (NOD EAE) I experiment 3 groups (n=10/group) • Vehicle • C60 (fullerene core without NMDA receptor antagonist) • ABS-75 II experiment 3 groups (n=9-10/group) • Vehicle • ABS-75 • Memantine B) SJL EAE 3 groups (n=15/group) • Vehicle • ABS-75 • Memantine C) Acute EAE C57BL/6J 2 groups(n=8/group) • Vehicle • ABS-75	A) NOD mice 10 weeks old B)SJL mice 8 weeks old	Immunohistochemistry Western Blot analysis Cytokines and T-cell proliferation assay Behavioral experiments (plus maze apparatus)	A)chronic progressive EAE (NOD EAE) Fullerene ABS-75 ↓ disease progression ↓ clinical score ↓ axonal loss ↓ myelin loss No impairment in memory functions No effect of ABS-75 was observed in the SJL and C57BL/6J relapsing models Memantine/MK-801 No effect on disease progression	A)chronic progressive EAE (NOD EAE) Fullerene ABS-75 No effect T, B-cell response ↓ oxidative stress ↓ CD11b infiltration ↓ CCL2 ↑ EAAT1 & GS (glutamine Synthetase) No effect of ABS-75 was observed in the SJL and C57BL/6J relapsing models,
Grasselli et al., 2013 (30)	CNQX (AMPA/kainate antagonist) 10 μM MK-801 6, 5 and 30 μM Tetrodotoxin (TTX) (voltage-dependent Na+ channel blocker) 1 μM 4-aminopyridine (4-AP) (K^+^ channel blocker) 100 μM Bicuculline (GABAa Blocker) 10 μM	Chronic Relapsing EAE Immunization of 200 mg myelin oligodendrocyte glycoprotein (MOG 35-55) in 300 mL of complete Freund’s adjuvant (CFA) containing 8 mg·mL-1 Mycobacterium Tuberculosis Pertussis toxin (500 ng) was injected on the day of the immunization and again 2 days later	Onset of clinical signs 20 dpi	Continuous intracranial infusions for 4 weeks Preclinical phase of EAE (7–9 dpi) acute (20–25 dpi) phase of EAE	3 main groups • Naïve • CFA • EAE Subgroups • EAE+MK-801 • EAE+MK-801+TTX • EAE+CNQX • EAE+4-AP • EAE+4-AP+TTX	Female C57BL/6 mice 6–8 weeks old also WT (Ddo+/+ knockout (KO, Ddo–/–) mice	Minipump implantation Electrophysiology Western blotting Immunohistochemistry Confocal microscopy NAD(P)H fluorescence imaging	MK801 ↓ delayed acute phase ↓ clinical score ↓ Clinical deficits ↓ synaptic transmission alterations ↓ sEPSC frequency (TTX co-incubation reversed this effect) ↑ PPR(paired pulse ratio) CNQX Fully abolished sEPSCs MK-801 & CNQX ameliorated NAD(P)H response in EE 4-AP ↑ ↑ sEPSC(co-incubation TTX blocked this effect) EAE mice (Ddo)with genetically enhanced NMDA signaling had opposite effects	
Chechneva et al., 2011 (32)	Dextromethorphan (DMX) (NMDA blocker, σ1 agonist, SERT/NET inhibitor) 0.1 and 10 mg/kg	Immunization s.c. in the flank with an emulsion of MOG35–55 peptide in incomplete Freund’s adjuvant supplemented with Mycobacterium tuberculosis H37Ra (M.t.). Using different concentrations of MOG/M.t., two types of EAE trials were initiated. EAE-I (severe) was induced with 300 μg of MOG and 2.5 mg/ml M.t., and EAE-II (moderate-Relapsing remitting MS) with 50 μg MOG and 0.5 mg/ml M.t. Mice also received two i.p. injections of 250 ng of pertussis toxin immediately after immunization and 48 h later	EAE-I(severe) Onset of clinical symptoms 10 dpi Peak 16–19 dpi EAE-II(moderate) Onset of clinical symptoms 10 dpi Peak 16–19 dpi	(i.p) once daily beginning on 7 dpi until 45 dpi	EAE-I(severe) n=22/group 3 groups • saline • DXM 0,1 mg/kg • DM 10 mg/kg EAE-II(moderate) n=22-23/group 3 groups • saline • DXM 0,1 mg/kg • DXM 10 mg/kg	C57/BL6 male mice 8–12 weeks old Weight 25–35 g	Real time qPCR Immunohistochemistry ROS production assay (lucigenin-enhanced chemiluminescence assay)	EAE-I(severe) *Statistically insignificant results* EAE-II(moderate) DXM 0.1 mg/kg) ↓ clinical score ↓ infiltration ↓ inflammation ↓ demyelination ↓ axonal loss	EAE-I(severe) *Statistically insignificant results* EAE-II(moderate) DXM 0.1 mg/kg) ↓ NOX2 expression ↓ superoxide (ROS) ↓ CD4&CD8 mRNA ↓ CD45& CD11b mRNA ↓ INF-γ mRNA ↓ IL-17 & CCL2 mRNA ↑ sigma-1 mRNA
Wang et al., 2021 (31)	(R-) Ketamine (GluN2B/NMDAR antagonist)10 mg/kg	Immunized with myelin oligodendrocyte glycoprotein (MOG)35–55 emulsified in complete Freund’s adjuvant (CFA) supplemented with Mycobacterium tuberculosis H37Ra	Onset 12–13 dpi	*Preventive treatment* i.p. to mice daily from 30 mins before the injection immunization to the 14th day after immunization (15 days)	3 groups (n=10/group) • Control (saline) • EAE+Saline • EAE+R-ketamine	Female C57BL/6 J mice 10–13 weeks old weight 19–22 g	Immunohistochemistry Immunofluorescence	↓ clinical score ↓ weight loss ↓ inflammatory cell infiltration ↓ demyelination ↓ microglial activation ↓ BBB damage	
Xia et al., 2021 (33)	2-Benzofuranyl-2-imidazoline (2-BFI) (NMDA inhibitor, imidazoline 2 receptor agonist) 20 mg/kg	Immunization with MOG35–55 emulsified in complete Freund’s adjuvant (CFA), which supplemented with 8 mg/ml mycobacterium tuberculosis H37Ra and was then administered subcutaneously to multiple sites in the fanks of each mouse (Then, mice were injected with pertussis toxin day 0 and 2 dpi	onset of clinical symptoms 10 dpi.	i.p.20 mg/kg 2-BFI twice daily for 14 days beginning on 10 dpi	4 groups(n=12/group) • saline–saline control • saline +2-BFI • EAE–saline • EAE+2-BFI	Female C57BL/6 mice weight 18–20g	BBB Permeability of Evans Blue Immunofluorescence Staining Immunohistochemistry Western Blot Analysis	↓ neurological deficits ↓ BBB permeability ↓ infiltration of inflammatory cells	↓ loss of occluding ↓ NR1-ERK pathway activation
Fazio et al, 2008 (34)	RO0711401 (mGlu1R positive allosteric modulator) 10 mg/kg MPEP 10 mg/kg SIB-1757 (mGlu5R negative allosteric modulators) 10 mg/kg JNJ16259685 (mGlu1R antagonist) 2.5 mg/kg	Immunization s.c. with 200 mg of MOG35–55 peptide emulsified in 0.1 ml of incomplete Freund’s adjuvant containing 2 mg M. tuberculosis. After immunization, 200 ng of pertussis toxin in 200 ml phosphate-buffered saline (PBS) was injected i.p. on the day of immunization and 2 days later. Sham-immunized mice receiving incomplete Freund’s adjuvant containing 2 mg M. tuberculosis without MOG35–55 followed by pertussis toxin were used as controls	*1^st^ set of experiment* Onset of clinical deficits 8–10 dpi Peak of disability 30 dpi. *2nd set of experiment* Onset of clinical deficits 8 dpi Peak of disability 20 dpi.	*1st set of experiment* i.p. administration at 15 or 30 dpi *(pretreatment &post-treatment)* 2^nd^ set of experiment i.p. administration at 10 or 20 dpi (*pretreatment &post-treatment)*	6 groups • Sham immunized mice(saline) • EAE mice • EAE+ RO0711401 • EAE+MPEP • EAE+ SIB-1757 • EAE+JNJ16259685 • (pretreatment &post-treatment)	C57BL/6 male mice (22–24 g, BW), 6–7 wks old	Western Blot Immunohistochemistry Rotarod test Paw print test	RO0711401 ↑ motor coordination ↓ motor deficits Improved motor symptoms JNJ16259685 ↓ motor performance	
Di Prisco et al., 2016 (35)	LY379268 (mGlu2/3 receptor agonist) 0.01 to 1 mg/kg LY341495 (mGlu2/3 receptor antagonist) 100 nM	Immunization s.c. with incomplete Freund’s adjuvant containing 4 mg/mL Mycobacterium tuberculosis (strain H37Ra) and 400 μg of the myelin oligodendrocyte glycoprotein 35–55 (MOG35–55) peptide, followed by i.p. administration of 250 ng of pertussis toxin on day 0 and after 48 h.	Disease onset 10–12 dpi	i.p. 3 h before being killed(21 dpi)	4 groups • Control • EAE • LY379268-treated control • LY379268-treated EAE	Mice C57BL/6J (female and male) 18–20 g, 6–8 wks-old	Immunoblotting Synaptosome release assay Immunocytochemical analysis Confocal microscopy	LY379268 ↓ clinical score	LY379268 ↓ ↓Glu release in cortical and spinal cord synaptosomes Inhibits Glu exocytosis
Besong 2002 (36)	L-2-amino-4-phosphonobutyric acid (L-AP-4) (mGluRIII agonist) 200 μl of 250 mM	Immunization with 50 μg of guinea pig MBP, 2 mg Mycobacterium tuberculosis in 100 μl saline, and 100 μl Freund’s incomplete adjuvant	Onset of clinical signs 10 dpi	Animals were implanted s.c. osmotic minipumps containing 200 μ l of 250 mM of L-AP-4 dissolved in PBS, which release 250 nl/d for 28 d. Control animals were implanted with minipumps containing PBS alone. Animals were immunized 48h later	3 groups(n=10/group) • Control • EAE+PBS • EAE+L-AP-4	Lewis rats Weight 225–250 g	Northern blot analysis Western blot analysis RT PCR *In vivo* microdialysis Immunohistochemisrty	↓ clinical score ↑ rate of recovery	↓ mRNA RANTES (cerebellum and spinal cord) ↓ MHC-II+(27 dpi) ↓ CD4+ cells (27 dpi)
Volpi et al., 2016 (37)	ADX88178 10, 30 and 60 mg/kg N-Phenyl-7- (hydroxyimino) cyclopropachromen-1a-carboxamide (PHCCC) (mGlur4 positive allosteric modulators) 3 mg/kg	*Relapsing-remitting EAE induction* SJL/J female mice were immunized with 100 mg of proteolipid protein peptide 139e151 (PLP139e151)emulsified in complete Freund adjuvant (CFA; with 5 mg/ml Mycobacterium tuberculosis (BD Difco). Each mouse s.c. injections of 200 ml emulsion, in draining axillary and inguinal lymph nodes. Pertussis toxin (PTX, 200 ng/mouse was administered i.p. on the day of immunization and 48 h later.	Onset of clinical signs 10–12 dpi	s.c.injections daily for the first two weeks and every other day for the third week, starting 10 days(onset)	5 groups • Vehicle • EAE+PHCCC • EAE+ADX881878–10 mg/kg • EAE+ADX881878–30 mg/kg • EAE+ADX881878–60 mg/kg	female SJL/J mice 10wks old C57BL/6 mice 8–12 wks old (*in vitro* exp)	Real-Time PCR Western blotting Skin test assay ELISA HPLC cAMP determination assay	ADX88178(30 mg/kg and 60 mg/kg) ↓ clinical score ADX88178 has long term therapeutic effects	ADX88178 and ADX104608 (negative control) ↑ IL-10, TGF-β ↑ IDO1 expression PHCCC ↓ cAMP levels in DC
Gammon et al., 2015 (38)	PHCCC 3 mg/kg soluble or NPs (polymeric nanoparticles)	Immunization with two (s.c.) injections of emulsions prepared from complete Freund’s adjuvant (CFA) and 100 μg of MOG35–55 in killed mycobacterium tuberculosis H37Ra (200–500 μg). Subsequently, 200 ng of pertussis toxin in 100 μL of PBS was injected intra-peritoneal (i.p.) on the day of immunization and again the following day.	Onset 10 dpi Peak of disease 14 dpi	Both soluble and NPs PHCCC administered s.c. at the tail base every 3 days or every 5 days beginning on the day of EAE induction	• Vehicle/control • EAE+Soluble PHCCC every 3 days • EAE+ NPs PHCCC every 3 days • Vehicle/control • EAE+Soluble PHCCC every 5 days • EAE+ NPs PHCCC every 5 days	C57BL/6 mice 10wks old	Flow cytometry ELISA Toxicity assays T-cells proliferation assay	NPs PHCCC ↓ clinical score ↓ disease severity Delayed disease onset	NPs PHCCC ↓ T-cell proliferation ↓ inflammatory cytokines ↓ Th17 cells ↑ T-reg cells ↓ DC activation

Results of *in vivo* papers classified by type and dose of agent, induction protocol and signs of EAE, drug administration, design of study, species, methods, clinical and biological results.

↓, Decrease:↑, Increase.

### 3.2 AMPA receptor agents in EAE

AMPA receptor antagonists, especially the quinoxalinedione family of compounds (QX compounds), constitute a pharmacological class extensively researched for their effects in EAE. The competitive AMPA/kainate antagonist NBQX demonstrated consistent efficacy across multiple studies. Pitt et al. showed that NBQX (300 μg) ameliorated clinical impairment, reduced dephosphorylation of neurofilament H, decreased axonal damage, and promoted oligodendrocyte survival in MBP-induced EAE in Lewis rats (39). These findings were confirmed by Werner et al., who failed to detect any significant anti-inflammatory properties of the compound (40).

Smith et al. conducted comprehensive comparisons of competitive (NBQX, MPQX) and non-competitive (GYKI52466, GYKI53773) AMPA antagonists in acute EAE. NBQX (30 mg/kg) and the more AMPA-selective MPQX (10 mg/kg) reduced disease severity, motor disability, disease duration, and neurological deficits. The non-competitive antagonists GYKI52466 and GYKI53773 (30 mg/kg) similarly attenuated neurological deficits. NBQX and MPQX did not inhibit concanavalin A–induced T-lymphocyte proliferation, proposing that their effects might be mediated through neuroprotection rather than immunomodulatory or anti-inflammatory mechanisms (41). These findings are largely supported by Groom et al., who conducted a broad evaluation of multiple AMPA antagonists (NBQX, MPQX, GYKI52466, GYKI53773, BIIR561, CP465022) using acute, adoptive transfer, and chronic EAE models. All antagonists demonstrated significant clinical benefit, including reduced neuronal loss, decreased peak neurological scores, shortened disease duration, and improved motor function. In contrast, aniracetam, a positive allosteric AMPA modulator, worsened disability and accelerated disease onset, providing further evidence for glutamate excitotoxicity in EAE pathophysiology. A notable difference from the results of Smith et al. was that non-competitive antagonists showed significant inhibition of T-cell proliferation, suggesting additional immunomodulatory mechanisms (42).

Further studies confirmed the efficacy of NBQX in reducing the clinical severity of EAE while adding further mechanistic insights. It has been demonstrated that NBQX (30 mg/kg) reversed the upregulation of the excitatory amino acid carrier 1 (EAAC-1), normalized the expression of glial glutamate transporters GLT-1 and GLAST, and restored plasma membrane calcium ATPase 2 (PMCA2) and collapsin response mediator protein 1 (CRMP1) levels (43, 44).

Combinatorial approaches have also been evaluated. Kanwar et al. assessed a combination of NBQX with an anti-MAdCAM-1 monoclonal antibody, with or without the NMDA antagonist GPE (6 mg/kg), as a neuroprotective strategy. This multimodal approach markedly improved physical features, alertness, motor function, and disease severity, while reducing a range of inflammatory biomarkers and rescuing neurons from apoptosis (45).

The above results are presented in **Table 2**.

**Table 2:** Comparative assessment of *in vivo* studies on the effects of glutamatergic (AMPA) agents on disease scores and progression.

Study	Drug and dosage	Induction of EAE protocol	Signs of EAE	Preventive or symptomatic treatment (drug administration)	Study design	(Species) age/gender/weight	Methods	Clinical results	Biological results
Pitt et al., 2000 (39)	NBQX (Competitive AMPA/Kainate antagonist) 300 μg in 200 μl PBS	Immunization with MBP. MBP was dissolved in sterile PBS at a concentration of 8 mg/ml and emulsified with an equal volume of incomplete Freund’s adjuvant supplemented with 6 mg/ml Mycobacterium tuberculosis	Onset of clinical signs 7–9 dpi	Treatment started on day 5 post immunization (sc) until day 15	2 groups • Vehicle(PBS) n=18 • NBQX n=19	Female adult SJL/J mice 4–6 weeks old	Western Blot analysis T-cell proliferation assay Immunohistochemistry	NBQX ameliorates clinical impairment	↓ dephosphorylation of neurofilament H ↓ axonal damage ↑ oligodendrocyte survival
Smith et al., 2000 (41)	NBQX (Competitive AMPA antagonist) 3–30 mg/kg MPQX (Competitive AMPA antagonists) 2.5–10 mg/kg GYKI52466 (Non-competitive AMPA antagonists) 30 and 40 mg/kg GYKI53773 (Non-competitive AMPA antagonists) 30 mg/kg	immunization (sc) in the dorsal surface of each hind foot with 50 μl inoculum containing 50 μg guinea pig myelin basic protein (MBP) and emulsified in Freund’s complete adjuvant containing 5.5 mg/ml Mycobacterium tuberculosis H37Ra ‘Sham-immunized’ rats received Freund’s complete adjuvant containing M. tuberculosis alone “subcutaneously	Onset of clinical signs(neurological decline) 11 dpi Peak 13 dpi 13–16 dpi (neuronal loss)	NBQX 3–30 mg/kg(i.p) twice daily for 7 days starting on day 10 dpi MPQX 2.5–10 mg/kg (i.p.) twice daily for 7 days starting on 10 dpi GYKI52466 40 mg/kg (i.p) at 0, 6, 12, 18 and 24 h on 10 dpi 30 mg/kg (i.p) twice daily for 7 days starting on 10 dpi. GYKI53773 30 mg/kg (i.p) twice daily for 7 days starting on 10 dpi.	NBQX 4groups • vehicle (n=10) • NBQX 3 mg/kg (n=10 • NBQX 10 mg/kg) (n=10) • NBQX 30 mg/kg) (n=30) • MPQX • 4groups • vehicle (n=26) • MPQX 2,5 mg/kg (n=10) • MPQX 5 mg/kg) (n=10) • MPQX 10 mg/kg) (n=12) GYKI52466/GYKI53773 3 groups • Vehicle(n=9) • GYKI52466–30 mg/kg(n=10) • GYKI53773–30 mg/kg(n=10)	Lewis rats weight 200–220 g	Immunohistochemistry Neurological assessment Quantification of neuronal density Quantification of neuroinflammation. Quantification of immunosuppression in an *in vitro* stimulation assay Electron microscopy	NBQX (30 mg/kg) ↓ disease severity ↓ motor disability and duration of disease ↓ neurological deficits in acute EAE MPQX (10 mg/kg) ↓ motor disability and duration of disease ↓ neurological deficits in acute EAE GYKI52466 and GYKI53773 (30 mg/kg) ↓ neurological deficits in acute EAE AMPA antagonists do not have anti-inflammatory or immunomodulatory properties.They are related with the involvement of glutamate in EAE pathogenesis	↓ neuronal death NBQX and MPQX did not inhibit the concanavalin A-induced proliferation of T lymphocytes
Kanwar et al., 2004 (45)	NBQX Coupled with anti-MAdCAM-1 mAb and neuroprotector GPE (NMDA antagonist) 6 mg/kg (50% ip and 50% iv)	Immunization(s.c) with 300 mg of myelin oligodendrocyte glycoprotein 35 ± 55 (MOG35 ± 55) peptide and emulsified in complete Freund’s adjuvant (CFA) containing 500 mg of Mycobacterium tuberculosis H37Ra 500 ng of pertussis toxin in 200 ml phosphate-buffered saline (PBS) injected i.v. via the tail vein, followed 48 h later by a second dose. A second injection of MOG peptide was given in the\ absence of pertussis toxin one week later	Onset of clinical symptoms 28 to 35 dpi Peak of disease (paralysis) 42–45 dpi	NBQX 6 mg (50% i.p.- 50% i.v.) Antibody (500 mg) in tail vein (70%) And i.p. (30%) at 10 mg/kg GPE 30 mg (50% i.p.- 50% i.v.)	5 groups(n=5/group) • NBQX • GPE • GPE+NBQX • anti-MAdCAM-1 mAb, • combination of NBQX-GPE- anti-MAdCAM-1 mAb) 5 in treatment group-5 in control group (neuropathology analysis)	C57BL/6 mice (8 ± 10 weeks old)	Immunohistochemistry Western blot analysis TUNE ELISA RNAse protection and anti-MOG35 ± 55 antibody assays Measurement of cell type specific apoptosis	improvements in physical features ↑ alertness, ↑ spontaneous mobility, ↑ motor function ↑ sensory function, ↑ weight shiny hair/skin firmness triple combination -> greatest improvement in the disability scores	↓ expression of proinflammatory and anti-inflammatory cytokines NBQX blocks glutamate/AMPA receptors expression ↓ neuron apoptosis ↓ cytokine accumulation in the CNS Combination therapy: ↓ IL-10 mRNA ↓ IL-4, IL-6, IL-10, IL-12, ↓ TNF-α and IFN-γ Only anti- MAdCAM-1 mAb blocked anti-MOG35 ± 55 antibody production
Ohgoh et al., 2002 (43)	NBQX 30 mg/kg	Immunization (sc) in each hind foot pad with a 50 μl inoculum containing 50 μg guinea pig myelin basic protein, emulsified in Freud’s complete adjuvant containing 5,5 mg/ml Mycobacterium tuberculosis H37Ra	Onset of clinical signs 11 dpi	NBXQ (30 mg/kg (i.p) twice a day for 5 days starting 7 dpi	3 groups • normal(n=6) • saline (n=6) • NBQX (n=6)	Lewis rats 7–9 weeks Weight 180-200g	Immunohistochemistry Western blot analysis Semi-quantitative PCR	Inhibition of the appearance of EAE symptoms	Reversal of increase of EAAC-1 protein in EAE Reversal of decrease expression of glial transporters (GLT-1 and GLAST) to normal levels
Kurnellas et al., 2010 (44)	NBQX 30 mg/kg	Immunization (s.c.) in the flank with an emulsion consisting of 200 mg myelin oligodendrocyte glycoprotein35–55 (MOG35–55 in saline and an equal volume of complete Freund’s adjuvant containing 7 mg/ml Mycobacterium tuberculosis H37RA. Intraperitoneal injection of 350 ng of pertussis toxin at 0 and 48 h post-immunization	??????	NBXQ (30 mg/kg (s.c.) daily a)24h after onset of symptoms(early intervention) b) at the peak of disease (late intervention)	3 groups • control (saline)(n=6) • EAE (vehicle)(n=6) • EAE + NBQX(n=6)	C57BL/6 mice, 9 to 10-week-old	Immunohistochemistry Western blot analysis MTT assay Mass spectrometry 2D electrophoresis	Prevention of progression of EAE and reversal of neurological deficits	Restoration of the decreased levels of PMCA2 and CRMP1
Groom et al., 2003 (42)	NBQX 3,10, 30 mg/kg MPQX 2.5, 5,10 mg/kg GYKI52466–30 mg/kg GYKI53773–30 mg/kg BIIR-561 (Non-competitive AMPA antagonist) 30 mg/kg CP465022 (Non-competitive AMPA antagonist) 10 mg/kg Aniracetam (positive allosteric AMPA modulator) 30 mg/kg	a) Active EAE model (Lewis rats) Immunization (s.c.) in the dorsal surface of each hind foot with 50µL inoculum containing 50µg purified guinea pig myelin basic protein, complete Freund’s adjuvant (CFA), and Mycobacterium tuberculosis H37Ra 5,5 mg/ml b) adoptive transfer EAE 10 days after immunization Lewis rats were sacrificed and spleens removed Splenic lymphocytes were cultured with purified guinea pig myelin basic protein at 1 μg/mL and were administered (i.p.) to non-immunized Lewis rats. c) Chronic relapsing EAE induced in Biozzi mice. Immunized s.c. on day 0 and day 7 in the flank at three sites with 0.3 mL of the emulsion (1 mg spinal cord homogenate, 60 μg of combined M. tuberculosis and butyricum) In addition, mice were injected i.p. with 200ng of pertussis toxin (Bordetella pertussis, Calbiochem; 2 μg/mL in phosphate buffered saline) immediately and 24h after immunization with neuroantigens	Onset of disease 9–11 dpi Peak 12–13 d Onset of disease on day 4 post transfer. Peak reached by day 6	a) Active EAE model NBQX, 3, 10, and 30 mg/kg i.p., and MPQX, 2.5, 5, and 10 mg/kg i.p., were given twice daily for seven days starting 10 (dpi) . GYKI52466, GYKI53773, BIIR561, and aniracetam were given 30 mg/kg i.p. twice daily, starting day 10 CP465022 was examined at a dose of 10 mg/kg i.p. twice daily b)adoptive transfer EAE NBQX, (30 mg/kg) i.p., or vehicle twice daily for seven days starting on day 4 after transfer. c) Chronic relapsing EAE 1) Acute phase NBQX, 30 mg/kg i.p., twice daily on 10–16 dpi 2)chronic phase NBQX 30 mg/kg i.p. or vehicle was given to Biozzi mice once daily between days 26 and 42 after immunization	a) EAE in Lewis Rats 4 groups (NBQX) • vehicle (n = 40) • NBQX, 3 mg/kg (n = 10) • NBQX 10 mg/kg (n=10) NBQX30 mg/kg (n =21). 4 groups (MPQX) • vehicle (n =26) • MPQX, 2,5 mg/kg (n = 10) • MPQX 5 mg/kg (n=10) –MPQX 10 mg/kg (n =16). 2 groups (GYKI52466) • vehicle (n = 14) • GYKI52466, 30 mg/kg (n =15) 2 groups (GYKI53773) • vehicle (n = 9) • GYKI53773, 30 mg/kg (n =10) 2 groups (BIIR561) • vehicle (n = 15) • BIIR561–30 mg/kg (n =16) 2 groups (CP465022) • vehicle (n = 15) • CP465022–10 mg/kg (n =16) 2 groups (aniracetam) • vehicle (n = 10) • BIIR561 (30 mg/kg (n =10) b)adoptive transfer EAE 2 groups • vehicle(n=8) • NBQX 30 mg/kg(n=8) c) EAE in Biozzi mice 2 groups • vehicle(n=10) • NBQX 30 mg/kg(n=10)	adult Lewis rats (active EAE, adoptive transfer) Biozzi mice Weight 20–22g (Chronic relapsing EAE)	Light Microscopy Electron Microscopy Immunohistochemistry	a) Acute EAE NBQX ↓ neuronal loss ↓ peak of neurological score ↓ duration of EAE ↑ motor function ↓ cumulative score ↓ weight loss MPQX (10 mg/kg) Dose dependent reduction of disability Delayed disease onset ↓ peak of neurological score ↓ duration of EAE ↓ cumulative score GYKI52466 ↓ peak of neurological score ↓ cumulative score GYKI53773 ↓ ↓ symptoms ↓ ↓duration of disability ↓ ↓ peak of neurological score BIIR561 ↓ weight loss ↓ peak of neurological score ↓ duration of disability ↓ cumulative score CP465022 ↓ peak of neurological score ↓ cumulative score Aniracetam -worsened disability Earlier onset of disease Extension of the duration of symptoms b)adoptive transfer EAE NBQX (30 mg/kg) ↓ ↓ peak and cumulative disease score c) EAE in Biozzi mice NBQX (30 mg/kg) ↓ disability ↓ clinical score	Inflammation NBQX Not significant reduction in inflammation Immunosuppression (GYKI52466,GYKI53773 BIIR561,CP465022) Significant Inhibition of T-cell proliferation
Werner et al., 2000 (40)	NBQX 300 ug in 200 ul PBS	Adoptive transfer EAE MBP was dissolved in sterile PBS (8 mg/ml) and emulsified with an equal volume of incomplete Freund’s adjuvant supplemented with 6 mg/ml Mycobacterium tuberculosis Ten days after injection of antigen into the flanks of SJL/J mice, lymph node cells (LNC) were obtained from draining lymph nodes, cultured and grown for 4 days in the presence of MBP (50~g/ml), and subsequently injected i.v. into syngeneic mice at a dose of 3 x 107 cells/mouse.	Onset of disease 7–9 d after transfer	NBQX three daily (s.c.) beginning on day 5 post transfer and continuing until the termination of the experiment (day 15)	3 groups • normal • EAE vehicle (PBS) • EAE + NBQX (300 μg/200 μl PBS)	Female adult (4–6 weeks old) SJUJ mice	T-cell proliferation assay Western blot analysis	↓ clinical score	↓ abnormally dephosphorylated NF-H ↓ loss of oligodendrocytes ↓ axonal damage Not significant anti-inflammatory properties

Results of *in vivo* papers classified by type and dose of agent, induction protocol and signs of EAE, drug administration, design of study, species, methods, clinical and biological results. ↓, Decrease: ↑, Increase.

### 3.3 Agents affecting glutamate synthesis, metabolism, and release in EAE

#### 3.3.1 Glutaminase and GCPII inhibitors

Glutamate carboxypeptidase II (GCPII) hydrolyzes the neuropeptide N-acetylaspartylglutamate (NAAG) into glutamate and N-acetylaspartate (NAA), while glutaminase catalyzes the conversion of glutamine into glutamate and ammonia, thereby increasing glutamate levels.

2-Phosphonomethyl pentanedioic acid (2-PMPA, 10 mg/kg), a GCPII inhibitor, was found to exert marked reductions in clinical scores, neuroinflammation, and disease severity, while delaying disease onset in MOG-induced EAE. Treatment decreased infiltration of CD4+ T-cells and CD11b+ macrophages, reduced mGluR1 expression and glutamate levels, attenuated Th1/Th17 cytokines (IL-17, IFN-γ, TNF-α), and reduced GCPII expression, with results suggesting that mGluR3 activation might be responsible for some of the observed effects (46). In other studies, higher doses of the compound (100–300 mg/kg) improved learning, memory, and cognitive performance in EAE mice, increased NAAG levels in the frontal cortex, and minimized weight loss, with cognitive benefits retained even in delayed treatment scenarios (47, 48).

Shijie et al. studied 6-diazo-5-oxo-L-norleucine (DON), a glutaminase inhibitor, and carbenoxolone (CBX), a gap junction inhibitor, in an MOG-induced EAE model. Treatment with either compound (0.016–1.6 mg/kg DON; 0.2–20 mg/kg CBX) reduced clinical scores, inflammatory cell infiltration, and peak disease severity, while delaying disease onset and rescuing neurons from death (49).

#### 3.3.2 Glutamate release inhibitors

Riluzole, an inhibitor of glutamate release used in the treatment of amyotrophic lateral sclerosis, is another compound studied for its effects in EAE. At doses of 10 mg/kg, it reduced disease development and clinical severity in EAE, with decreased axonal damage, demyelination, inflammation, and leukocyte transmigration, while also activating the TREK-1 potassium channel and upregulating ICAM1, VCAM1, and PECAM1 mRNA (50, 51).

Higher doses of riluzole (100 μg/mL, oral administration) have been tested in both prophylactic and chronic treatment paradigms. Reductions in CNS glutamate levels and clinical scores were observed, along with increased Foxp3 and GFAP protein expression and decreased immune-related markers. However, chronic treatment alone did not significantly reduce clinical scores, highlighting the importance of treatment timing (52).

The above results are presented in **Table 3**.

**Table 3:** Comparative assessment of *in vivo* studies on the effects of agents affecting synthesis/metabolism/release of on disease scores and progression.

Study	Drug and dosage	Induction of EAE protocol	Signs of EAE	Preventive or symptomatic treatment (drug administration)	Study design	(Species) age/gender/weight	Methods	Clinical results	Biological results
Shijie et al., 2009 (49)	6-diazo-5-oxo-L-norleucine (DON) (glutaminase inhibitor) 0,016, 0,16 and 1.6 mg/kgCarbenoxolone CBX (gap junction inhibitor) 0.2, 2 and 20 mg/kg	Immunization (s.c.) at the base of the tail with 0,2 ml of emulsion containing 200 μg MOG_33–35_ peptide in saline combined with equal volume of incomplete Freund’s adjuvant containing 300 μg heat-killed *Mycobacterium tuberculosis* H37Ra. Mice were injected with 200ng pertussis toxin i.p. on the day of immunization and 2 days after	Onset 10 dpi Peak of disease 14–20 dpi	DON or CBX i.p. every other day beginning from day of immunization and fro 5 wks	2 EXP/4 groups • EAE+PBS • EAE+DON 0,016 • EAE+DON 0,16 • EAE+DON 1,6 • EAE+PBS • EAE+CBX 0,2 • EAE+CBX 2 • EAE+ CBX 20	C57BL/6 mice 6–8 wks old	Histological analysis Clinical score assessment	↓ clinical score ↓ inflammatory cell infiltration ↓ peak of disease Delayed onset	↓ Glu release (activated microglia) ↓ neuronal death ↓ inflammatory cell infiltration
Ha et al 2016 (46)	2-phosphonomethyl pentanedioic acid (2-PMPA) (GCPII inhibitor) 10 mg/kg LY341495 (mGluR3antagonis) 1 mg/ml	Immunization (s.c.) with 200 lg of MOG35-55 peptide in complete Freund’s adjuvant (CFA) containing 500 lg of heat-inactivated Mycobacterium tuberculosis on day 0 supplemented by intravenous injection of 200 ng of pertussis toxin on day 2	Onset of clinical signs 9–10 dpi Peak (13 dpi)	2-PMPA i.p. twice a week from the day of EAE induction LY341495 (i.p) 30 min before either 2-PMPA or PBS injection.	a)Lewis rats 3 groups • Naïve • peak (13d p.i.) • recovery (19d p.i) b) C57BL/6 mice 2 groups (n=10-13/group) • Vehicle(PBS) • 2-PMPA • 4 groups • vehicle • LY341495 • 2-PMPA • LY341495 + 2-PMPA	Female C57BL/6 mice 6–7 weeks old weight 18–22 g female Lewis rats 8–12 weeks old	Immunohistochemistry Immunofluorescent staining ELISA Western Blot analysis RT-PCR Flow cytometry	2-PMPA ↓ clinical score ↓ neuroinflammation ↓ disease severity Delayed disease onset	2-PMPA ↓ infiltration of T-cells/macrophages(CD4+, CD11b+) ↓ mGluR1 expression ↓ Glu level ↓ Th1/Th17 cytokines(IL-17, INF-γ, TNF-α) ↓ GCPII expression Activated mGluR3
Rahn et al, 2012 (47)	2-PMPA 100 mg/kg 2-PMSA (GCPII inhibitor) 100 mg/kg	Immunization s.c. with incomplete Freund’s adjuvant containing 4 mg·mL 1 Mycobacterium tuberculosis (strain H37Ra) and 400 μg of the myelin oligodendrocyte glycoprotein 35–55 (MOG35–55) peptide, followed by i.p. administration of 300 ng of pertussis toxin on day 0 and after 48 h.	Onset of clinical signs 10 dpi Peak of disease 20 dpi	EXP I (i.p) daily 100 mg/kg PMPA2 or PMSA2 from the day of EAE induction EXP II Administer vehicle, PMPA2 or PMSA2–30 min before behavioral testing	EXP I 3 groups • Control + vehicle (saline) • EAE+vehicle • EAE+MPMA2 • EXP II • Control + vehicle (saline) • EAE+vehicle • EAE+MPMA2 • EAE+PMSA	7- 8-wk-old female C57BL/6 mice	Barnes Maze. Fear Conditioning Elevated Plus Maze. Mass spectrometry Perfusion and FACS Immunostaining GCPII Activity Assay	2-PMPA ↑ learning & memory ↑ cognitive performance	2-PMPA ↑ NAAG(frontal cortex)
Hollinger et al 2016 (48)	2-PMPA 10, 30, 100, and 300 mg/kg	Immunization s.c. with incomplete Freund’s adjuvant containing 4 mg·mL 1 Mycobacterium tuberculosis (strain H37Ra) and 400 μg of the myelin oligodendrocyte glycoprotein 35–55 (MOG35–55) peptide, followed by i.p. administration of 300 ng of pertussis toxin on day 0 and after 48 h.	Onset of clinical signs 10–11 dpi	EXP I (i.p) daily 10,30, 100, 300 mg/kg PMPA2 from the day of EAE induction EXP I (Delayed treatment) (14 dpi) (i.p) 100 mg/kg MPMA-2	EXPI 5 groups (n=7-11/group) • EAE+Vehicle (Hepes) • EAE+ PMPA 10 mg/kg • EAE+PMPA 30 mg/kg • EAE+PMPA 100 mg/kg • EAE+PMPA 300 mg/kg EXPII Delayed treatment 2 groups (n=12-14/group) • EAE+Vehicle • EAE+100 mg/kg	7–8 week old female C57BL/6 mice	Elevated plus maze Barnes maze Mass spectrometry	EXP I PMPA-2 (100 mg/kg)improves cognition PMPA-2(300 mg/kg)reduced weight loss EXP II Delayed treatment improves cognitive impairment Delayed 2-PMPA treatment does not affect EAE disease score or body weight	EXP I 2-PMPA causes a dose-dependent increase in brain NAAG EXP II Delayed treatment ↑ NAAG (brain)
Gilgun-Sherki et al 2003 (50)	Riluzole (Glu release inhibitor) 10 mg/kg	Immunization (sc)with the peptide encompassing amino acids 35–55 of rat MOG (200 ml emulsion containing 75 mg MOG peptide (300 mg in the case of C57/bl) in complete Freund’s adjuvant (CFA) and 200 mg Mycobacterium tuberculosis	Onset of clinical signs 12 dpi	Symptomatic treatment (twice a day, i.p.) Group II (Riluzole d 12-13) Group III (Riluzole d18-27) Group IV (Riluzole d 12-27)	4 groups, 10 per group Group I saline Group II (Riluzole d 12-13) Group III (Riluzole d18-27) Group IV (Riluzole d 12-27)	6–8 weeks old C3H.SW/C57/female mice Weight 20g	Histological analysis (H&E, LFB Bielshowesky’s silver) Immunohistochemistry T-cell proliferation assay	Reduction in disease development and clinical severity	↓ axonal damage ↓ demyelination ↓ inflammation
Bittner et al., 2013 (51)	Riluzole 10 mg/kg	Immunization with MOG35–55 peptide supplemented with complete Freund’s adjuvant (CFA) to obtain a 1 mg ml−1 emulsion, subcutaneously at the flanks of deeply anesthetized mice. Pertussis toxin was injected intraperitoneally on the day of immunization and then again 2 d later at a dose of 400 ng (	Onset of clinical signs 10 dpi	i.p. administration from the day of immunization or when the mice showed the first clinical signs of EAE.	2 groups control(untreated) WT EAE+RIL	6- to 8-week-old female C57BL/6 or Kcnk2−/− mice	Flow cytometry TER measurements Western blot analysis Transmigration assays RT-PCR Electrophysiological measurements DHE staining. Nitrotyrosine staining Transwell permeability assay Determination of nitric oxide and ROS production	↓ EAE clinical score	↑ TREK-1 activation ↓ leukocyte transmigration ↑ mRNA expression of ICAM1, VCAM1 and PECAM1 ↓ immune cell infiltration
Rotolo et al., 2021 (52)	Riluzole 100 μg/mL (p.o. admin)	Immunization (s.c.) with 100 μg myelin oligodendrocyte glycoprotein peptide fragment 35–55 (MOG) emulsified in incomplete Freund’s adjuvant supplemented with 500 μg Mycobacterium tuberculosis H37RA. Approximately 50 μL of emulsion was injected into each hind flank (0.1 mL total). All mice including controls were given 200 ng of intraperitoneal pertussis toxin (i.p.) on days 0 and 2 to activate the immune system and enhance EAE development. This model of EAE induction is non-relapsing and is considered a chronic, sustained form of EAE.	Onset of clinical signs 12 dpi	a) Prophylactic +chronic treatment (21 days prior EAE induction) EAE + 25 μg/mL RIL EAE + 100 μg/mL RIL b) chronic treatment (on day 0 of EAE induction) - EAE + 100 μg/mL RIL	a) Prophylactic +chronic treatment 4 groups (n = 10/group). Control, EAE, EAE + 25 μg/mL RIL EAE + 100 μg/mL RIL b) chronic treatment 3 groups (n = 10/ group). Control, EAE, EAE + 100 μg/mL RIL	female C57BL/6 mice 14 weeks old	Hot plate test Histological analysis Western Blot analysis	a) Prophylactic +chronic treatment ↓ Glu in CNS tissue ↓ clinical score b) chronic treatment no significant reduction in clinical score	a) Prophylactic +chronic treatment ↑ expression of Foxp3 protein ↑ expression of GFAP protein in astrocytes ↓ spleen weight ↓ immune related markers (T-reg, CD45, CD4)

Results of *in vivo* papers classified by type and dose of agent, induction protocol and signs of EAE, drug administration, design of study, species, methods, clinical and biological results.

↓, Decrease:↑, Increase.

### 3.4 Agents affecting glutamate transporters in EAE

#### 3.4.1 Glutamate transporter expression enhancers

Laquinimod (1.25 mg/kg), a compound that enhances glutamate transporter expression, was found to reduce clinical symptoms and motor impairments during acute EAE. Treatment increased Slc1a3 mRNA, which encodes the GLAST transporter, as well as glial GLT-1 transporter levels, thereby minimizing EAE-associated glutamate alterations (53).

Ceftriaxone, a β-lactam antibiotic known to boost EAAT2 transcription, (200 mg/kg) alleviated tactile allodynia and cognitive deficits, restored novel object recognition in EAE mice, and was associated with increased EAAT2 expression and reduced NMDA receptor phosphorylation (54). Similarly, Melzer et al. confirmed that ceftriaxone (200 mg/kg) lowered clinical scores and disease severity by reducing T-cell proliferation and infiltration, as well as decreasing proinflammatory cytokines such as IFN-γ and IL-17 (55).

Olechowski et al. also tested MS-153, a GLT-1 glutamate transporter activator (10 mg/kg), which lowered FOS expression but did not significantly affect pain behavior or nociceptive responses in EAE mice (56).

#### 3.4.2 Xc^–^ transporter inhibitors

Xc^–^ transporter inhibitors, such as sulfasalazine (SAS), are substances known to block the cystine–glutamate antiporter, thereby decreasing extracellular glutamate concentrations.

Sulfasalazine (SAS; 0.15 g/kg) and S-4-carboxyphenylglycine (S-4-CPG; 0.010 g/kg), another inhibitor of the Xc^–^ transporter, were evaluated in MOG–induced EAE in C57BL/6 mice. In the chronic version of the EAE model, SAS significantly reduced clinical disease scores, demyelination, reactive gliosis, and inflammation, accompanied by decreased T-cell infiltration and lower expression of inflammatory mediators (NF-κB, IL-17, and IFN-γ) (57). In the relapsing–remitting EAE model, SAS similarly decreased clinical disease severity and reactive gliosis. S-4-CPG produced comparable reductions in clinical scores and demyelination in the chronic EAE model.

#### 3.4.3 Na^+^/Ca^2+^ (NCX) exchange inhibitors

Rossi et al. examined bepridil and KB-R7943, Na^+^/Ca^2+^ exchange inhibitors, using *in vitro* electrophysiological approaches. Bepridil (100 μM) or KB-R7943 (10 μM) treatment modulated evoked excitatory postsynaptic currents (eEPSCs) and paired-pulse ratio (PPR) in striatal slices from EAE mice (58). Bepridil produced different effects depending on the disease phase. PPR increases were noted during the acute phase, whereas eEPSCs decreased, while a reversal of these observations was observed during the chronic phase. KB-R7943 showed similar acute-phase effects, increasing PPR while reducing eEPSCs.

#### 3.4.4 Indirect modulators of glutamate transport

Phenelzine (15 mg/kg), a monoamine oxidase inhibitor, demonstrated delayed onset of clinical signs, normalized mechanical thresholds, and antinociceptive effects in EAE. Treatment also normalized presynaptic excitatory synaptic densities, reduced VGLUT1 reactivity, decreased cortical microgliosis, and restored neuronal morphologies (59).

Blockade of the IL-1 receptor by IL-1ra (150 ng/day) resulted in reduced clinical scores, motor deficits, and inflammation, with delayed disease onset in MOG-induced EAE, probably by normalizing GLAST/EAAT1 expression levels and reducing eEPSCs (60).

The above results are presented tabularized in **Table 4**.

**Table 4:** Comparative assessment of *in vivo* studies on the effects of agents affecting glutamate transporters on disease scores and progression.

Study	Drug and dosage	Induction of EAE protocol	Signs of EAE	Preventive or symptomatic treatment (drug administration)	Study design	(Species) age/gender/weight	Methods	Clinical results	Biological results
Gentile et al., 2018 (53)	Laquinimod (Glu transporter expression enhancer) 1.25 mg/kg	Immunization s.c. with 200 g (MOG35-55) in 300 l of complete Freund’s adjuvant (CFA) containing Mycobacterium tuberculosis (8 mg/ml, strain H37Ra and emulsified with PBS. All animals were injected with 500 ng of pertussis toxin intravenously on the day of immunization and 2 days later	Onset 10 dpi	One week before immunization, s.c. implantation with osmotic minipumps and intracerebroventricular (icv) infusion of either vehicle (vhl) or laquinimod (1.25 mg/kg/day) for 4 weeks	2 groups EAE+vehicle EAE+laquinimod	female mice, C57BL/6N 7–8 wks old	Osmotic minipumps Electrophysiology Western blot qPCR Immunofluorescence Confocal microscopy	↓ clinical score ↓ motor disability (acute phase)	↑ Slc1a3 mRNA coding for GLAST transporter ↑ glial GLT-1 transporter ↓ glutamate alterations
Olechowski et al., 2013 (54)	Ceftriaxone (b-lactam antibiotic EAAT 2 transcription enhancer) 200 mg/kg	immunization (s.c.) with 50 μg of MOG35–55 emulsified in complete Freund’s adjuvant (CFA). CFA was supplemented with an additional 5-mg/ml heat killed Mycobacterium tuberculosis H37Ra to a final concentration of 6 mg/ml. The final concentration of CFA in the emulsion was 1.5 mg/ml. An intraperitoneal (I.P.) injection of 300 ng Pertussis toxin (Bordatella pertussis) (Sigma-Aldrich, Oakville, ON) was administered at the time of induction and again 48 h later	Disease onset 13–14 dpi	200 mg/kg ceftriaxone i.p. daily 7 dpi	4 groups CFA+vehicle(n=5), EAE+vehicle(n=10) CFA+ceftriaxone n=5, EAE+ceftriaxone(n=10)	10–12 week old female C57BL/6 mice	Behavioral testing Mechanical allodynia Novel object recognition (NOR) test General activity/attention score Rotarod assay Molecular methods Real-time RT-PCR Western blot analysis	↓ tactile allodynia and cognitive deficits Restores novel object recognition (NOR)	↑ EAAT2 expression ↓ NMDAR phosphorylation
Melzer et al., 2008 (55)	Ceftriaxone 200 mg/kg	Immunization with (MOG35–55).Mice were injected subcutaneously with 200 mg of MOG35–55 peptide emulsified with complete Freund’s adjuvant containing Mycobacterium tuberculosis (5 mg/ml). Control animals were immunized with an equivalent volume of CFA emulsion that did not contain MOG35–55 peptide.All mice were injected i.p. with pertussis toxin (400 ng; List Biological Laboratories, Campell, CA, USA) at the time of immunization and 2 days later	Disease onset 10–11 dpi	(i.p.) either from the day of immunization (MOG+CTX permanent)) or from the individual onset of symptoms (MOG+CTX therapeutic	3 groups (n=8/group) MOG(saline) MOG+CTX permanent MOG+CTX therapeutic	Female WT C57BL/6 mice 6–8 weeks old	Western blot analysis Assessment of T-cell invasion T-cell activation & antigen-recall assay Immunophenotyping	↓ clinical score ↓ disease severity	↓ T-cell proliferation ↓ T-cell infiltration, migration(CD4+) ↓ proinflammatory INFγ, IL-17
Olechowski et al., 2010 (56)	MS-153 (R)--1-methyl-1- nicotinoyl-2-pyrazoline (GLT-1 Glu transporter activator) 10 mg/kg LY341495 0.25 mg/kg	Immunization s.c. with 50 lg of MOG35–55 emulsified in complete Freund’s adjuvant. The CFA was supplemented with an additional 4-mg/ml heat killed Mycobacterium tuberculosis H37Ra to a final concentration of 5 mg/mL. An intraperitoneal injection of 300 ng Pertussis toxin was administered at the time of induction and again 48 h later. Control mice were treated with CFA and Pertussis toxin alone	Onset 15–16 dpi Peak of disease 19 dpi	i.p. 20 min prior formalin testing	3 experiments/2 groups (I) CFA, (n=5) EAE(n=6)+ MS-153 (i.p. 20 min prior formalin testing) (II) CFA, (n=3) EAE(n=3) + MS-153 (i.p. and euthanasia 1h later) (III) CFA, (n=5) EAE(n=6)+ LY341495 (i.p. 20 min prior formalin testing)	female C57BL/6 mice 10–12 wks old	Formalin test Immunocytochemistry Western blot Motorod test	No significant differences in pain behavior, nociceptive responses,	MS-153 ↓ FOS expression
Evonuk et al., 2015 (57)	sulfasalazine (SAS) (Xc-transporter inhibitor) 0.15 g/kg [(S)-4-carboxyphenylglyc ne (S-4-CPG) (Xc-transporter inhibitor) 0.010 g/kg	a) Male C57Bl/6 mice 50 μg MOGp and 125 μg desiccated Mycobacterium tuberculosis H37Ra emulsified in incomplete Freund’s adjuvant as well as intraperitoneal injections of 200 ng Bordetella pertussis toxin in PBS on days 0 and 2 b)female SJL mice (relapsing-remitting EAE) g 150 μg PLP139–151 and 500 μg desiccated Mycobacterium tuberculosis H37Ra emulsified in incomplete Freund’s adjuvant c) C3H/HeSnJ and C3H/HeSnJSlc7a11sut/sut mice Immunization with 100 μg PLP190-209 and 400 μg desiccated Mycobacterium tuberculosis H37Ra emulsified in incomplete Freund’s adjuvant as well as intraperitoneal injections of 400 ng Bordetella pertussis toxinin PBS on days 0 and 2	Onset of clinical signs 10 dpi	a)Male C57Bl/6 i.p twice.daily beginning 7 dpi b) SJL mice (relapsing-remitting EAE i.p twice.daily beginning 24 dpi	a)Male C57Bl/6 2 groups EAE+PBS(n=20) EAE+SAS(n=19) 2 groups EAE+PBS(n=5) EAE+S-4CPG(n=5) 3 groups Control(n=3) EAE+PBS(n=4) EAE+SAS(n=3) b) SJL mice (relapsing-remitting EAE 2 groups EAE+PBS(n=8) EAE+SAS(n=8)	Male C57Bl/6 mice 8–10 wks old female SJL mice 9 wks old C3H/HeSnJ and C3H/HeSnJSlc7a11^sut/sut^ mice 9 wks old females	Immunocytochemistry Flow cytometry Nitric oxide assay Glutamate assay Immunoblotting Perfusion Luxol fast blue staining Confocal microscopy Fluorescent Microscopy	a)Male C57Bl/6 SAS ↓ clinical score ↓ demyelination ↓ reactive glia ↓ inflammation (S-4-CPG) ↓ clinical score ↓ demyelination b) SJL mice (relapsing-remitting EAE SAS ↓ clinical disease score ↓ reactive glia	SAS ↓ T-cell infiltration ↓ NF-kB ↓ IL17+, INF-γ+, Foxp3+ ↓ Xc expression(elevated in EAE)
Rossi et al., 2010 (58)	Bepridil (Na+/Ca++ exchange inhibitors) 100 μM in ACSF KB-R7943 (Na+/Ca++ exchange inhibitors) 10 μM in ACSF Tetrodotoxin (TTX) (blocker of voltage-dependent Na+ channels) 1 μM in ACSF	Immunization (s.c.) with 300 ll of 200 lg MOG (35–55) in incomplete Freund’s adjuvant containing 8 mg ml1 Mycobacterium tuberculosis (strain H37Ra).Pertussis toxin (500 ng) was injected on the day of the immunization and again 2 days later	Onset 10–15 dpi Presymptomatic phase 7 dpi Peak of disease 20–25 dpi (acute phase) chronic phase of disease (50 dpi)	Dilution in ACSF	4groups Control(25 dpi) EAE +Bep(7 dpi)_ EAE+Bep(25 dpi) EAE+Bep (dpi) Control(25 dpi) EAE +KB-R(7 dpi)_ EAE+KB-R(25 dpi) EAE+KB-R (dpi) (25 dpi) Control EAE+(Bepridil+TTX)	Female C57BL/6 mice 6–8 wks old	Electrophysiological assessment Whole-cell patch clamp	a) Bepridil Acute phase ↑ PPR ↓ eEPSC Chronic Phase ↑ eEPSC ↓ PPR b)KB-R7943 Acute phase ↑ PPR ↓ eEPSC c) Bepridil+TTX Acute phase ↑ eEPSC frequency	
Potter et al, 2018 (59)	Phenelzine (MAO-i) 15 mg/kg	Subcutaneous 50 μg MOG 35-55	Onset of clinical signs day 14–17 dpi	Treatment onset 7 days after immunization. Daily (IP) injection of either vehicle or phenelzine (15 mg/kg).	IHC analysis I)control (CFA) II)vehicle(VEH)+EAE III)PLZ+EAE	8-12wk old Female C57/BL6	Rotorod assay FA imaging (FAI) Von Frey hair assay (mechanical sensitivity) Histological analysis Golgi-Cox staining Immunohistochemistry (IHC)	delayed onset of clinical signs of EAE Chronic PLZ normalized mechanical thresholds in EAE demonstrated antinociceptive effect	Normalized pre-synaptic excitatory synaptic densities in S1 ↓ VGLUT1+ density (↓ VGLUT1 reactivity) normalized cortical Iba-1+ reactive microglial cells in S1 (↓ excessive cortical Glu release, ↓ cortical microgliosis) normalized neuronal morphologies
Mandolesi et al, 2013 (60)	IL-1ra (IL-1 receptor antagonist) 150 ng/day	Immunization s.c. with 200 g (MOG35-55) in 300 l of complete Freund’s adjuvant (CFA) containing Mycobacterium tuberculosis (8 mg/ml, strain H37Ra and emulsified with PBS. All animals were injected with 500 ng of pertussis toxin intravenously on the day of immunization and 2 days later	Onset 10 dpi Peak 20 dpi	icv infusion for 4 weeks in EAE and CF	3 groups Vehicle (CFA)+ IL-1ra (n=16) EAE EAE+IL-1ra (n=17)	Female C57BL/6 mice 7–8 wks old	Minipump implantation Electrophysiology ELISA assay Real time PCR Western Blot t Bromodeoxyuridine labeling Immunohistochemistry Confocal microscopy	↓ clinical score ↓ motor deficits ↓ inflammation Delayed disease onset	↓ ePSC Normalization of GLAST/EAAT1 expression levels

Results of *in vivo* papers classified by type and dose of agent, induction protocol and signs of EAE, drug administration, design of study, species, methods, clinical and biological results.

↓, Decrease:↑, Increase.

## 4 Discussion

Disease-modifying therapies (DMTs) transformed the way we manage MS by reducing relapse rates and delaying disability progression. That said important limitations persist that undermine their long-term effectiveness and hence patient outcomes. Although a broad array of DMTs exists for relapsing forms of MS, there are notable evidence gaps such as a lack of robust head-to-head comparisons and long-term follow-up data. This complicates optimal therapy selection and comparative effectiveness assessments for everyone involved (61). Furthermore, many DMTs target only peripheral immune mechanisms and have limited efficacy in non-relapsing progressive forms of MS. This is mirrored in recent late-stage clinical trials for therapies in primary and secondary progressive MS, that have yielded mixed results underscoring the challenge in addressing MS beyond inflammation. These gaps are made more complex by symptomatic “wearing-off” between doses of monoclonal antibody therapies, which can lead to worsening fatigue, motor dysfunction, and cognitive symptoms affecting quality of life and treatment adherence (62).

On the concept of adherence, these alongside tolerability issues further constrain the utility of DMTs. Factors such as treatment side effects, complex administration schedules, and patient perceptions of benefit are consistently associated with suboptimal persistence, worsening clinical outcomes and increasing healthcare burden (63). Other factors that complicates treatments is the heterogeneity of MS pathophysiology and the absence of reliable biomarkers predicting individual response. Thus we should keep in mind that current DMTs do not fully prevent progression or address underlying neurodegenerative mechanisms, particularly in progressive disease stages (64). Collectively, these limitations point to an urgent need for more personalized treatment strategies, improved long-term comparative research, and therapies with mechanisms of action that more directly target neurodegeneration and disease progression across all MS phenotypes.

Alongside a immune-mediated inflammatory mechanisms, a central role emerges for glutamate dysregulation and excitotoxicity in MS. Proton magnetic resonance spectroscopy (¹H-MRS) studies have demonstrated increased glutamate and glutamine concentrations in acute lesions and normal-appearing white matter of patients with MS, correlating with markers of axonal injury and disability progression (65, 66). Excess extracellular glutamate overstimulates ionotropic NMDA and AMPA receptors expressed on neurons and oligodendrocytes. This leads to pathological calcium influx, mitochondrial dysfunction, oxidative stress, and ultimately cell death a process known as excitotoxicity (39, 40). Inflammation through activated microglia and infiltrating immune cells can further enhance glutamate release while impairing astrocytic uptake via excitatory amino acid transporters (EAAT1/2) which worsens excitotoxic injury (67, 68). This concept of “immunoexcitotoxicity” provides a mechanistic bridge between inflammation and progressive neuroaxonal loss in MS.

In this already complex picture, glutathione also plays a role. Reduced glutathione levels detected in cortical and deep grey matter structures of patients with relapsing–remitting MS have been associated with disrupted glutamate metabolism and cognitive impairment, suggesting that oxidative imbalance amplifies glutamate-mediated toxicity (66, 69). Chronic neurodegeneration can also be linked to reactive astrocyte subtypes that downregulate glutamate transporters, effectively increasing extracellular glutamate accumulation (70). Further evidence is provided by EAE models where pharmacological modulation of glutamatergic signaling can attenuate axonal damage and clinical severity, supporting a causal role for glutamate dysregulation in disease progression (39). Taken together, these data indicate that glutamate-mediated excitotoxicity is not merely a secondary epiphenomenon of inflammation but a key contributor to tissue injury and progression in MS. This renders adjunctive neuroprotective strategies targeting glutamatergic pathways possibly clinically relevant.

**Figure 1:**
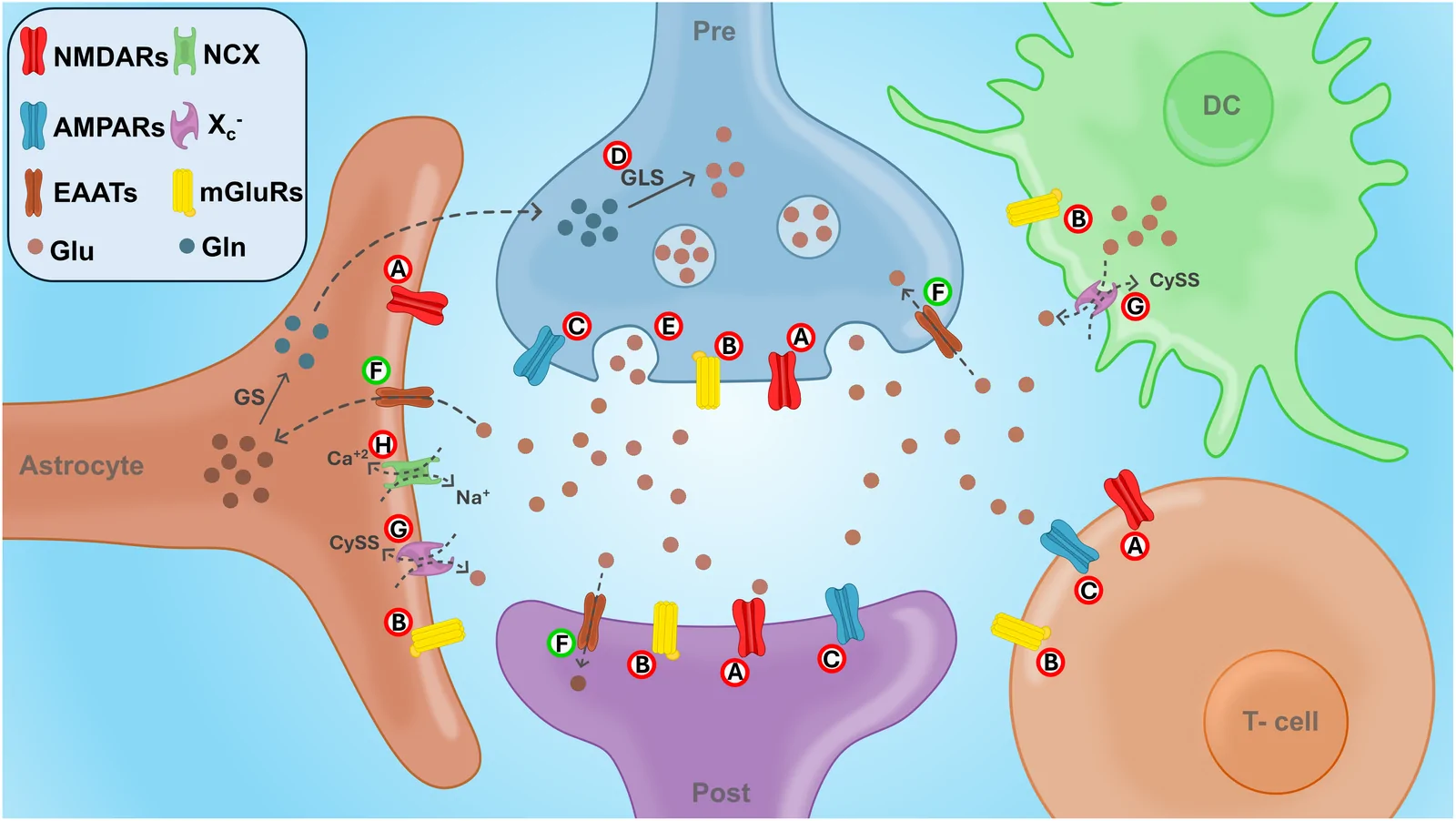
Mechanistic overview of glutamatergic signaling and pharmacological intervention targets in experimental autoimmune encephalomyelitis and multiple sclerosis. In neuroinflammatory conditions such as EAE and MS, dysregulation of glutamate homeostasis leads to excitotoxic signaling that contributes to both neuronal injury and immune cell activation. The figure depicts the tripartite glutamatergic synapse comprising the presynaptic terminal, postsynaptic compartment, and astrocyte extended to include dendritic cells and T cells, which express functional glutamatergic receptors and transporters that modulate their inflammatory activity. Under physiological conditions, glutamate released from the presynaptic terminal activates ionotropic receptors (NMDARs and AMPARs) and metabotropic receptors (mGluRs) on postsynaptic neurons and on immune cells. Astrocytes limit extracellular glutamate accumulation through EAATs and convert glutamate to glutamine via GS; glutamine is shuttled back to the presynaptic terminal, where GLS reconverts it to glutamate, completing the glutamate - glutamine cycle. System xc^–^ exchanges extracellular cystine for intracellular glutamate, contributing to extracellular glutamate levels, while NCX participates in ionic homeostasis and intracellular calcium regulation downstream of receptor activation. Circled letters indicate the principal sites of action of the pharmacological classes discussed in the review, each targeting a distinct node of this signaling network to reduce excitotoxic or pro-inflammatory glutamatergic activity: **(A)** NMDAR antagonists; **(B)** mGluR negative modulators; **(C)** AMPAR antagonists; **(D)** glutaminase inhibitors; **(E)** glutamate release inhibitors; **(F)** EAAT expression enhancers; **(G)** system xc^–^ inhibitors; **(H)** NCX inhibitors. Red arrows indicate inhibitory drug actions; green arrows indicate enhancing drug actions. Abbreviations: Glu, glutamate; Gln, glutamine; CySS, cystine; GS, glutamine synthetase; GLS, glutaminase; EAATs, excitatory amino acid transporters; NMDARs, N-methyl-D-aspartate receptors; AMPARs, α-amino-3-hydroxy-5-methyl-4-isoxazolepropionic acid receptors; mGluRs, metabotropic glutamate receptors; NCX, Na^+^/Ca^2+^ exchanger; DC, dendritic cell.

The available findings support glutamatergic dysregulation as a major mechanistic contributor to EAE pathophysiology and as a plausible contributor to MS pathobiology. A range of pharmacological interventions aimed at modulating various aspects of glutamatergic neurotransmission were reported, in EAE, to reduce neurological disability, attenuate disease severity and, in some studies, modulate inflammatory and oxidative parameters (**Tables 1**–**4**). The principal molecular targets of these interventions, spanning presynaptic glutamate release, ionotropic and metabotropic receptor activation, astrocytic reuptake, glutamine recycling, and cystine/glutamate exchange, are schematically represented in **Figure 1**, which also illustrates the glutamatergic machinery present in DCs and T cells as immune components relevant to EAE pathobiology (**Figure 1**). (5, 8, 37, 71–85). These effects were nevertheless model-, dose- and timing-dependent and have not, to date, been reproduced in human MS trials.

Within the NMDA receptor antagonist class, several agents (memantine, amantadine, MK-801 and GluN2B-selective antagonists)demonstrated reductions in clinical scores, disease progression, BBB permeability, and inflammatory infiltration (19–26, 28–30, 86). Two structurally related agents, memantine and amantadine exhibited clinical improvement concurrently with reductions in proinflammatory cytokines (IL-1β, IL-6, TNF-α) and chemokines, decreased oxidative stress, and preservation of antioxidant enzyme activity (SOD1) (22, 24). These findings suggest that NMDA antagonism may interrupt immunoexcitotoxicity. Another thing to note is that not all “NMDA antagonism” is made equal. It seems to be context-depended as shown by variability across dosing regimens and disease models in which the antagonists appear effective (27). One, here cannot help but notice particularly the absence of efficacy at lower doses or in certain chronic or relapsing paradigms. It is plausible that NMDA receptor overactivation is most prominent during acute inflammatory phases characterized by robust glutamate release and BBB disruption, whereas progressive stages may involve additional mechanisms less responsive to broad NMDA blockade. The different effects subunit-selective antagonists further indicate that receptor subunit composition may determine pathological versus physiological signaling, justifying a shift toward subtype-selective strategies to minimize off-target effects and increasing therapeutic effect (26, 86).

AMPA receptor antagonists demonstrated a more consistent neuroprotective efficacy. Competitive and non-competitive antagonists (NBQX, MPQX, GYKI compounds) reduced peak clinical severity, axonal damage, and disease duration across acute, adoptive transfer, and chronic EAE models (39–42). If the effects are mediated mostly through direct immunomodulation or neuroprotection, is still debated. It appears that AMPA blockade in some cases confers mostly neuroprotection than direct immunosuppression (41). Other studies identified partial inhibition of lymphocyte activation, hinting at potential model-specific immunomodulatory effects (42). Mechanistically, AMPA antagonists preserved neuronal integrity via normalizing glutamate transporter expression (GLT-1, GLAST), restoring calcium homeostasis regulators (PMCA2), and mitigating cytoskeletal injury (43, 44). As expected from the above, a positive AMPA modulator such aniracetam worsened disease parameters further strengthening the correlation between excessive AMPA receptor activation and EAE severity (42).

Metabotropic glutamate receptor modulation revealed a more complex and subtype-dependent profile. Evidence points to postsynaptic mGluR signaling being protective depending on the group of receptors that is being affected (21, 23–25, 34). While Group I mGluR antagonists (LY367385, MPEP) showed limited clinical efficacy despite molecular changes in myelin-related gene expression, positive modulation of mGlu1 improved motor coordination and clinical scores. Group II (mGlu2/3) and Group III (mGlu4) receptor activation consistently reduced disease severity and exerted robust immunomodulatory effects (35–38). These agents decreased Th1/Th17 responses, reduced dendritic cell activation, enhanced regulatory T-cell populations, and increased anti-inflammatory mediators such as IL-10, TGF-β, and IDO1. Because mGlu2/3 and mGlu4 receptors are predominantly presynaptic autoreceptors and inhibit glutamate release, their activation may simultaneously lessen excitotoxicity and shift immune system towards tolerogenic phenotypes. suggests that pharmacokinetic optimization may influence preclinical performance, although its translational relevance at the moment remains uncertain (38).

Additional mechanistic insight is provided by intervention aiming glutamate synthesis, trafficking and clearance. GCPII inhibition with 2-PMPA reduced inflammatory infiltration, Th1/Th17 cytokines, and glutamate levels while increasing NAAG concentrations implicating NAAG-mediated mGluR3 activation in neuroprotection (46–48). In a similar fashion, glutaminase inhibition (DON) and gap junction blockade (CBX) attenuated disease severity and neuronal death (49). Riluzole, a glutamate release inhibitor, reduced demyelination, axonal loss, and leukocyte transmigration though chronic monotherapy demonstrated limited efficacy, once again highlighting the importance of timing (50–52). Laquinimod and ceftriaxone, two drugs enhancing glutamate transporter expression, ameliorated clinical severity while restoring EAAT2/GLT-1 and GLAST levels (53–55). This finding supports the concept that impaired astrocytic glutamate clearance might contribute to sustained extracellular glutamate accumulation in EAE. The notion that CNS damage could be amplified by pathological glutamate release from immune and glial cells is also supported by Xc^–^ transporter inhibition (57). Sulfasalazine, an inhibitor of the aforementioned transporter, appears to reduce demyelination and inflammatory signaling.

Integrating the four agent classes discussed in the experimental tables with the clinical-trial outcomes in **Table 5** allows a possible unifying framework in which glutamatergic dysregulation in EAE/MS is presented as a self-reinforcing immunoexcitotoxic loop with phase-dependent therapeutic windows. In the early, peripherally driven inflammatory phase of EAE (characterized by BBB opening, infiltration of activated CD4+ T-cells and dendritic cells, and a sharp rise in extracellular glutamate from microglial system xc^–^ activity, impaired astrocytic uptake and synaptic spillover) the most reproducible benefit is obtained with agents that blunt the acute excitotoxic surge at the postsynaptic level. In this window AMPA receptor antagonists (NBQX, MPQX, GYKI compounds) show the most consistent neuroprotective signal across acute, adoptive-transfer and chronic EAE paradigms, by means of reducing axonal damage, oligodendrocyte loss and clinical disability with only partial immunomodulation (39–42). Broad and subunit-selective NMDA receptor antagonists could be considered similarly effective but with a markedly narrower therapeutic window and clear dose- and model-dependence (19–26, 28–31). In the transition to the subacute and chronic phases, when sustained extracellular glutamate accumulation is driven more by glial and immune sources than by acute synaptic release, agents acting on glutamate supply and clearance gain mechanistic prominence: system xc^–^ inhibition (sulfasalazine, S-4-CPG), EAAT2/GLT-1 transcriptional enhancement (ceftriaxone, laquinimod), glutaminase blockade (DON) and GCPII inhibition (2-PMPA) all reduce disease severity while engaging immune compartments, restoring astrocytic glutamate clearance, reducing Th1/Th17 cytokines, attenuating dendritic-cell activation and limiting CD4+/CD11b+ infiltration (46–49, 53–57). Modulation of metabotropic glutamate receptors integrates these two arms by simultaneously dampening presynaptic glutamate release and reshaping the immune phenotype: mGlu2/3 (LY379268) and Group III mGluR (L-AP-4, ADX88178, PHCCC) activation reduce clinical scores, expand regulatory T cells, increase IL-10/TGF-β/IDO1 and suppress dendritic-cell maturation, producing a coherent neuroprotective–immunoregulatory signal (35–38). This phase-dependent reading of the evidence is consistent with the timing-sensitive efficacy of riluzole, where prophylactic exposure was protective but chronic monotherapy was not (50–52), and with the failure of riluzole, lamotrigine, memantine and amantadine when administered in established progressive MS (87–91) (85, 87, 90, 92, 93). It also identifies presynaptic glutamate release, system xc^–^ activity and astrocytic EAAT capacity as the mechanistic nodes most likely to remain pharmacologically relevant in the chronic, microglia-driven progressive phase, and supports a shift in trial design toward agents and patient strata that match the dominant glutamatergic mechanism active at the time of intervention.

**Table 5:** Seminal clinical trials (completed or ongoing) investigating the drug repurposing of glutamatergic agents for Multiple Sclerosis (MS).

NCT number	Title/name	Compound	Status	Key findings
NCT01910259	MS-SMART Trial	Riluzole 50 mg BD	COMPLETED (445 patients)	NEGATIVE: No effect on brain atrophy vs placebo at 96 weeks. No clinical benefit on EDSS or disability (87).
NCT00257855	Lamotrigine for Neuroprotection in SPMS	Lamotrigine 400 mg/day	COMPLETED (120 patients)	NEGATIVE on primary outcome: No difference in brain volume loss. MIXED: Reduced deterioration of the timed 25-foot walk (p=0.02) (88).
NCT00300716	Memantine for Cognitive Impairment	Memantine 10 mg BD	COMPLETED (82 patients)	NEGATIVE: No improvement in PASAT or memory tests vs placebo (94).
NCT00638833	Memantine Therapy for MS (Spain)	Memantine 30 mg/day	TERMINATED (60 patients expected – terminated at 19)	ADVERSE: Trial stopped due to reversible neurological impairment (89).
NCT01074619	EMERITE Trial	Memantine 20 mg/day	COMPLETED (90 patients)	NEGATIVE: No difference in cognitive scores vs placebo (p=0.88). Poor tolerability (95).
NCT03436199	INROADS Trial	ADS-5102 extended release Amantadine at daily doses of 137 mg or 274 mg	COMPLETED (558 patients)	POSITIVE/MIXED: Improvement in walking speed although clinically not that relevant (91).
NCT03185065	TRIUMPHANT-MS Trial	Amantadine 100 mg BD	COMPLETED (141 patients)	NEGATIVE: Not superior to placebo for MS fatigue. More adverse events than placebo (90).
NCT05809414, EudraCT: 2021-004868-95	FETEM Trial	Amantadine 100 to 200 mg + TMS	UNKNOWN (144 patients estimated)	ONGOING: Evaluating amantadine and/or TMS for MS fatigue. Results pending (97).
NCT00050232	AVP-923 for PBA in MS Patients	Dextromethorphan/Quinidine	COMPLETED (96 MS patients)	POSITIVE for PBA: Significant reduction daily PBA episode rate (p<0.0001). Improved quality of life. Symptom treatment only (98).
NCT03500289	Ketamine Pilot for MS Fatigue	Ketamine 0.5 mg/kg IV	COMPLETED (18 patients)	POSITIVE: Significant reduction in MFIS at day 28 (p=0.04). Effect lasted 4 weeks. Well tolerated (100).
NCT05378100	INKLING-MS Trial	Ketamine IV infusions	RECRUITING (110 patients estimated)	ONGOING: Phase 2 trial at Johns Hopkins. Testing 1–2 ketamine infusions for MS fatigue. Based on positive pilot data (101).

EDSS, Expanded Disability Status Scale; MFIS, Modified Fatigue Impact Scale; PASAT, Paced Auditory Serial Addition Test; PBA, Pseudobulbar Affect; TMS, Transcranial Magnetic Stimulation.

**Figure 2:**
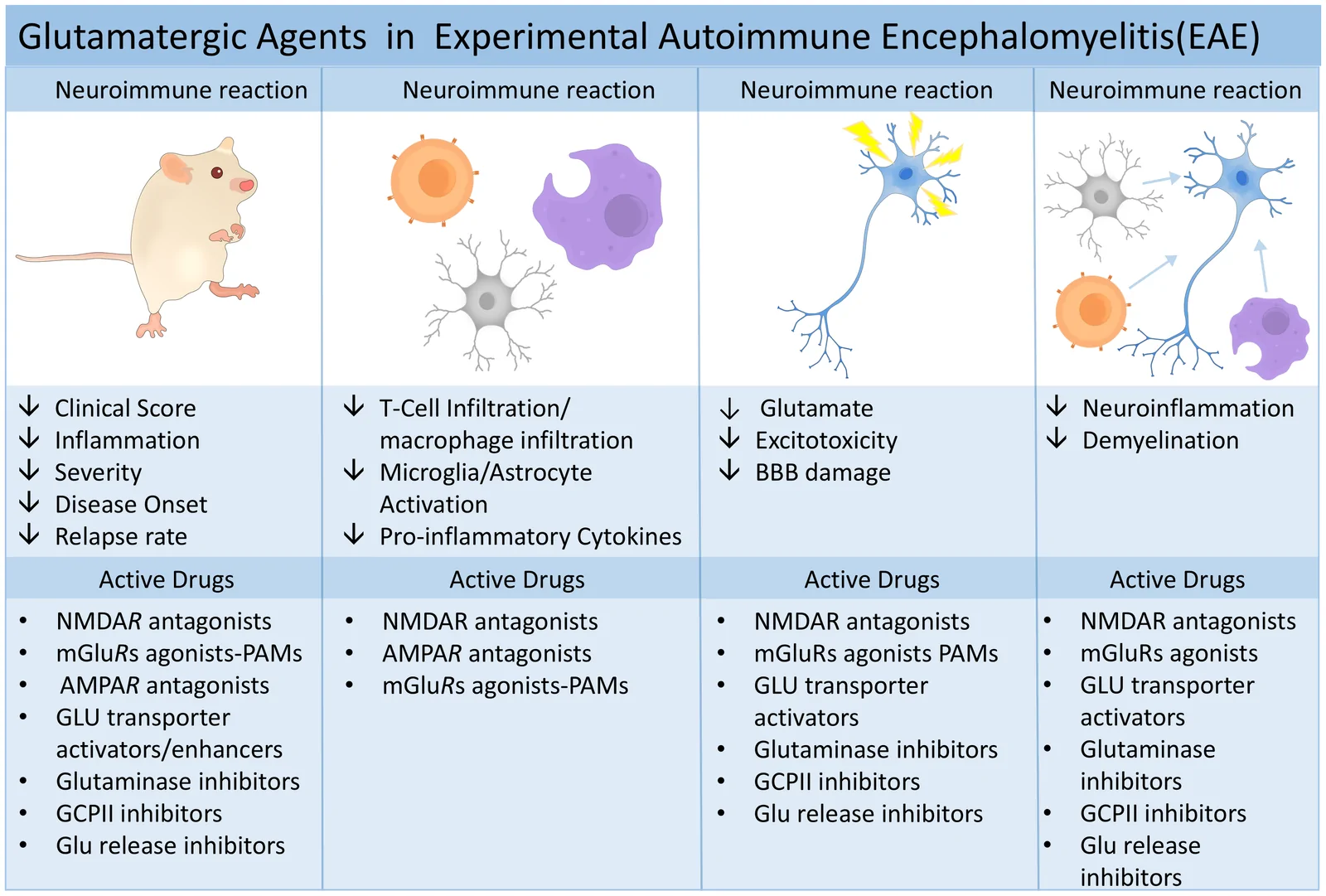
Glutamatergic agents in experimental autoimmune encephalomyelitis. Their effects on clinical markers, excitotoxicity, immune system cells and neuroimmune interactions.

Taken together, these findings point toward a multidimensional model in which glutamate dysregulation operates at several interconnected levels: (i) excessive synaptic and extrasynaptic receptor activation, (ii) impaired astrocytic uptake, (iii) enhanced metabolic production, and (iv) pathological release from activated immune and glial cells. This network forms a self-reinforcing excitotoxic–inflammatory loop that accelerates axonal degeneration and demyelination. “Surgically” modulating the glutamatergic system produces a measurable, mechanism-consistent benefit in currently used EAE models (**Figure 2**).

### 4.1 Clinical trials focusing on clinically approved glutamatergic agents

Among the glutamatergic agents evaluated so far in EAE models, some of them have already got clinical indications established, a fact that makes them attractive for drug repurposing. Specifically riluzole, memantine, amantadine, dextromethorphan, lamotrigine and ketamine have either completed or ongoing clinical trials that are summarized in **Table 5**.

Riluzole, the only approved disease-modifying therapy for amyotrophic lateral sclerosis (ALS) is a drug that inhibits glutamate release (92). This specific compound was trialed in the MS-SMART trial alongside fluoxetine and amiloride in a phase 2b, multiarm, double-blind, randomized placebo-controlled trial recruiting 445 patients with secondary progressive MS (87). No significant differences were observed in the primary outcome of MRI-derived percentage brain volume change (PBVC), a measure of brain atrophy, nor in secondary endpoints such as expanded disability status scale (EDSS) and disability progression.

Another drug tested in MS clinical trials is lamotrigine, an antiepileptic and mood stabilizer prescribed in epilepsy and bipolar disorder. While its action is not primarily on glutamate system, since it is categorized as a sodium channel blocker, it is known to also inhibit glutamate release (93). It has been trialed in 120 patients with secondary progressive multiple sclerosis to evaluate its effects on cerebral volume and walking speed (88). The drug failed to show benefit for cerebral volume, actually causing early volume loss that partially reversed on discontinuation, while it provided modest results for reduced deterioration of the timed 25-foot walk.

Memantine, a non-competitive NMDA receptor antagonist already approved for moderate-to-severe Alzheimer’s disease, is another drug that has been extensively trialed for disease-modifying properties in MS. In fact, it is the most studied glutamatergic drug in MS, with three clinical trials to date (89, 94, 95). These trials collectively enrolled 191 patients and tested dosages ranging from 10 mg twice daily to 30 mg/day, focusing mainly on cognitive markers, quality of life, fatigue, and depression. None showed improvement in these outcomes, and all reported higher rates of neuropsychiatric side effects in the treatment arm versus placebo. Of them the highest-dosage trial was terminated early, managing to recruit only 19 of 60 expected patients, due to reversible neurological impairments (89).

Amantadine, the parent molecule from which memantine is derived, is another non-competitive NMDA antagonist used in clinical practice for treating Parkinson disease and Influenza A (96). Clinical trials involving amantadine and MS focus mostly on MS related fatigue and walking speed. Currently there are 3 studies in total, two completed and one ongoing (90, 91, 97). The TRIUMPHANT-MS Trial, involving 141 patients, revealed that amantadine does not outperform placebo in treating MS-related fatigue (90). the study also assessed modafinil and methylphenidate, which were ineffective as well. The largest clinical study examining an extended release version of amantadine, the INROADS Trial involving 558 patients, demonstrated favorable outcomes of this amantadine formulation on walking speed (91). However the magnitude of effect did not warrant further clinical development and the company decided to abandon further development. Last but not least there is an ongoing trial studying amantadine with and without transcranial magnetic stimulation on MS-related fatigue and is estimated to have 144 patients (97).

A DXM-quinidine combination, which pairs an NMDA antagonist with a cytochrome P450 2D6 (CYP2D6) inhibitor, has been trialed in 18 MS patients to treat pseudobulbar affect for which it is already indicated (98). Although the sample was small, the treatment appeared to provide alleviation of PBA episodes. Here it should be mentioned that beyond its effects on glutamate, DXM exhibits rich pharmacological activity by also affecting serotonergic and noradrenergic neurotransmission (99).

Finally, two clinical trials have investigated ketamine, a relatively new addition to clinical armamentarium against depression, as a symptomatic treatment for MS-related fatigue (100, 101). The first was a promising pilot study involving 18 patients that demonstrated a significant reduction in MFIS scores. Building on those data, the ongoing INKLING-MS trial is currently recruiting an estimated cohort of 110 participants.

All in all, as it becomes evident from the above, the preclinical results come in stark contrast with the sobering reality of clinical trials. The clinical trials show low translatability of the results acquired through the EAE model with any relief observed symptomatic at best. As it stands, the compounds discussed in this review remain far from meeting the criteria for DMTs.

### 4.2 Translational limitations from bench to bedside

A well-known fact it is that while EAE models represent an invaluable *in-vivo* tool for studying multiple sclerosis, they can be quite distinct compared to MS as is found in humans (16, 102, 103). There are multiple models employed to study MS, each one reproducing only some aspects of this complex disease (16). Depending on the model, EAE is induced using different methods, and the resulting clinical symptoms and pathological features may be specific to the induction protocol employed. Given that different laboratories might employ different protocols this may hinder the reproducibility/translation of findings across laboratories using different methodologies (17). Standardization amongst research groups could be difficult also because different MS forms require different modeling approaches (16). Even if a standardization was possible, inter-species differences can limit translation efficacy.

There are important differences between the human and murine immune systems, both in terms of the immune cell populations central to the disease and the mechanisms underlying MS initiation and progression (16, 104). Another important consideration is disease onset and timing of treatment (104). In humans, disease onset typically occurs subclinically, and diagnosis and subsequent treatment often comes with a delay. In contrast, in EAE, treatment can be initiated at much earlier stages of disease development or even administered prophylactically.

Beyond inter-laboratory variability, several structural features of EAE constrain how its outcomes should be read against human MS, and these constraints are particularly relevant to the interpretation of glutamatergic interventions. First, classical EAE appears to be mostly a CD4+ Th1/Th17-driven disease induced by peripheral immunization against MHC class II-restricted myelin epitopes, whereas MS lesions also feature prominent CD8+ T-cell infiltration, meningeal B-cell aggregates and intrathecal antibody production (16, 102). Glutamatergic agents that act in part through modulation of CD4+ effector responses, for example mGluR4 positive allosteric modulators, GCPII inhibitors and the non-competitive AMPA antagonists with documented anti-proliferative effects on T-lymphocytes, may therefore engage a larger proportion of the relevant immunopathology in EAE than in MS. Second, most rodent EAE models are dominated by spinal cord and optic nerve inflammation, with limited cerebral and cerebellar cortical involvement, while cortical demyelination is a major contributor to disability and cognitive decline in MS (12, 102). This anatomical mismatch is particularly important for glutamatergic targets because NMDA and AMPA receptor subunit composition, EAAT distribution and excitotoxic vulnerability differ between spinal cord and cortical neurons; an agent shown to preserve spinal cord axons in EAE will not necessarily protect cortical neuronal networks in MS. Third, the EAE models most commonly used to evaluate glutamatergic agents are acute or acute-onset chronic, and most closely approximate RRMS rather than the slowly evolving, compartmentalized, microglia-driven neurodegeneration that characterizes progressive MS (16, 103). The acute inflammatory phase of EAE is accompanied by pronounced BBB disruption, infiltration of activated immune cells and a transient glutamate surge: precisely the conditions under which NMDA and AMPA antagonism appear most effective. Mapping this window onto progressive MS, in which BBB disruption is limited and excitotoxic injury is chronic and lower-grade, helps to explain why repurposed glutamatergic drugs that were beneficial in acute EAE have not produced disease-modifying effects in trials enrolling patients with established progressive disease (87, 88, 90, 91). Taken together, the immunological, anatomical and temporal mismatch between EAE and MS must be ingrained into the apparently positive preclinical signal of essentially every drug class discussed in this review.

At this point it would be useful to focus on the fact that EAE is characterized by an acute, peripherally driven inflammatory phase with prominent disruption of the BBB, conditions in which NMDA and AMPA antagonism appear most effective against (19–26, 28–30, 38, 40–42). The principal trials of glutamate modulators in MS, on the other hand, such as MS-SMART (riluzole) and the SPMS lamotrigine trial, enrolled patients with long-standing secondary progressive disease and minimal radiological inflammatory activity. MS-SMART investigators explicitly mention that exclusive targeting of axonal pathobiology is an inadequate strategy to achieve neuroprotection in progressive disease (87). This may suggest that interventions that display some success in acutely inflamed EAE tissue might not translate fully to compartmentalized, microglia-driven neurodegeneration as seen in chronic MS. A further interesting finding is the unexpected early reduction in cerebral volume that partially reversed on withdrawal reported in the lamotrigine trial. The drug produced an unexpected early reduction in cerebral volume (pseudoatrophy) that partially reversed on withdrawal. This was attributed to fluid shifts caused by the drug rather than to true neurodegeneration, highlighting how outcome measures derived from EAE histopathology might not always map onto MRI-based clinical endpoints in a straightforward manner (88). It must though be noted that disease-stage mismatch does not appear to be the whole story: a randomized phase II trial of riluzole in early MS also failed to reduce brain atrophy progression or to improve secondary endpoints (105). This argues against a simple “tested too late” fallback statement and strengthens the notion that glutamatergic agents face a broader efficacy problem in MS.

Apart from the above, the predicted validity of EAE itself must be taken into account. The recent systematic review and meta-analysis of 497 animal studies covering 26 approved or failed MS DMTs by Berg et al. reported no association between animal study outcomes, including clinically analogous EAE scores and neuroimaging metrics, and successful regulatory approval. This indicates that preclinical apparent efficacy is on its own a weak indicator of clinical benefit (17). Adding to the above a meta-analysis of 1117 EAE intervention studies by Vesterinen et al. showed that experiments reporting randomization or blinding produced significantly smaller effect sizes than those that did not. This suggests that the published preclinical efficacy of many neuroprotectants might partly be inflated by methodological bias and selective reporting (18). Ultimately, studies like those referenced above indicate that EAE literature is good for initial hypothesis testing, but it’s a poor predictor of which glutamatergic strategies will actually pass clinical trials.

Shifting the focus from the model to the intervention, the drugs to be tested encompass a diverse array of pharmacological mechanisms. NMDA receptor antagonists, AMPA receptor blockers, mGluR4 positive allosteric modulator, glutamate transporter enhancer, to name a few. However, only a limited number of agents from these classes have been evaluated in clinical trials. There is an evaluation bias towards those with existing clinical indications and therefore greater potential for drug repurposing. To complicate matters more, there is great pharmacodynamic heterogeneity even within a pharmacological category. For example some drugs in the NMDA receptor antagonist category, like DXM, lack mechanistic specificity whereas others more selective such as memantine and ketamine are not interchangeable (106–108). As far as the latter case is concerned, there are differences in the actions of ketamine and memantine. Ketamine in contrast to memantine inhibits the phosphorylation of eukaryotic elongation factor 2 and augments expression of BDNF (106). Ketamine and memantine also differentially desensitize NMDA receptors and probably due to pharmacokinetic differences cause different behavioral effects (107, 108).

A final constraint is intrinsic to glutamate pharmacology. Because NMDA and AMPA receptors mediate physiological synaptic transmission, plasticity and oligodendrocyte signaling, the therapeutic window between successful excitotoxicity blockade and disruption of normal function is narrow. It has been argued by Lipton et al. that early high-affinity NMDA antagonists failed clinically primarily because their tolerated doses failed to reach neuroprotective thresholds without producing unacceptable neuropsychiatric side effects (109). This is consistent with the early termination of the Spanish memantine trial after seven of nine actively treated patients developed transient blurred vision, fatigue, weakness and gait instability at 30 mg/day, symptoms which fully reversed on discontinuation of the drug in question (89). The same pattern is well documented outside the context of MS: NMDA receptor antagonists repeatedly showed neuroprotective promise in animal models of stroke and traumatic brain injury but failed in clinical trials, This appears to be in large part because dose-limiting psychotomimetic and cardiovascular effects prevented neuroprotective exposures from being reached in patients (110). Such observations may suggest that unfortunately the doses required to mimic the EAE-protective effect of memantine, and of NMDA antagonists more generally, in humans might be in the range that interferes with physiological glutamatergic signaling in a therapeutically unacceptable manner. Even in cases where the drug is not to be administered daily, see the example of ketamine as an antidepressant, one of the hurdles of repurposing the drug were side-effects such as dissociation, hallucinations and impaired cognition (111).

## 5 Conclusions and future directions

Glutamate neurotransmission plays a well-documented role in MS pathobiology, which has motivated substantial preclinical interest in its therapeutic modulation. Encouraging results from EAE studies prompted the initiation of clinical trials using already approved glutamatergic agents. In line with broader concerns about EAE-to-MS translation, those trials have so far largely failed to translate into clinical benefit. The MS-SMART trial failed to demonstrate riluzole’s efficacy on brain volume, while the TRIUMPHANT-MS and INROADS trials failed to demonstrate amantadine’s efficacy on MS fatigue and walking impairment respectively (90, 91, 97). While the translational failures can be disheartening, they can be treated as lessons on which a modified approach can be built.

Looking forward, there are many interesting and possibly rewarding research directions to pursue.

A first direction could be further characterizing and most importantly validating neurochemical biomarkers such as CSF glutamate levels (112). Biomarkers would not only give us another criterion for monitoring disease progress, but could also be used to stratify populations enrolled in clinical studies. Here glutamate measurements by means of proton MR-spectroscopy could distinguish patients into categories based on their glutamatergic profile (113–117). In the same line of thought, in combination with glutamate MRS measurements, investigation of genetic variants associated with MS and specifically with the glutamatergic system could contribute to the above (118).

Another direction would be revisiting MS models, as these serve as the primary platforms for initial drug testing and development. While we owe much of our molecular understanding of the disease to current models, they are not without limitations. On the subject of *in vivo* models, non-murine primates like marmosets can have clinical similarities to human MS and hence offer high translational value (103, 119). Given the ethical and logistical challenges of primate research, an alternative could be using humanized murine models (16, 120, 121). Rats and mice genetically engineered to express human-specific MS components could yield results closer to human pathophysiology. In *In-vitro* drug screening, human CNS cells derived from patient-specific induced pluripotent stem cells (iPSCs) can be integrated with 3D culture methods, such as spheroids and organoids, or co-culture platforms that simulate the complex interplay between microglia and astrocytes (122–125) These advanced *in vitro* models incorporate the human-specific pathophysiology of MS and enable the study of cellular interplay and spatial dynamics that are typically absent in standard 2D cultures.

Lastly, a deeper understanding of the structural and functional specifics of the glutamatergic receptors and enzymes could benefit further development of drugs (126–128). Future drug development can shift toward taking advantage of ligand-biased signaling and subunit-specific allosteric modulation. Such finely-tuned agents could selectively trigger the desired intracellular pathways while bypassing the off-target effects or dose-limiting toxicities. Here, artificial intelligence and machine learning can provide an advantage by leveraging predictive modeling and virtual compound screening (129, 130).
